# Genetic Screens Identify Additional Genes Implicated in Envelope Remodeling during the Engulfment Stage of Bacillus subtilis Sporulation

**DOI:** 10.1128/mbio.01732-22

**Published:** 2022-09-06

**Authors:** Helena Chan, Najwa Taib, Michael C. Gilmore, Ahmed M. T. Mohamed, Kieran Hanna, Johana Luhur, Hieu Nguyen, Elham Hafiz, Felipe Cava, Simonetta Gribaldo, David Rudner, Christopher D. A. Rodrigues

**Affiliations:** a Australian Institute for Microbiology and Infection, University of Technology Sydney (UTS), Sydney, Australia; b Department of Microbiology, Unit Evolutionary Biology of the Microbial Cell, Institut Pasteur, Paris, France; c Hub Bioinformatics and Biostatistics, Department of Computational Biology, Institut Pasteur, USR 3756 CNRS, Paris, France; d Laboratory for Molecular Infection Medicine Sweden (MIMS), Department of Molecular Biology, Umeå University, Umeå, Sweden; e Department of Microbiology, Harvard Medical School, Boston, Massachusetts, USA; f School of Life Sciences, University of Warwick, Coventry, United Kingdom; National Institute of Child Health and Human Development (NICHD)

**Keywords:** sporulation, engulfment, peptidoglycan, peptidoglycan remodeling, cell envelope, morphogenesis, spores

## Abstract

During bacterial endospore formation, the developing spore is internalized into the mother cell through a phagocytic-like process called engulfment, which involves synthesis and hydrolysis of peptidoglycan. Engulfment peptidoglycan hydrolysis requires the widely conserved and well-characterized DMP complex, composed of SpoIID, SpoIIM, and SpoIIP. In contrast, although peptidoglycan synthesis has been implicated in engulfment, the protein players involved are less well defined. The widely conserved SpoIIIAH-SpoIIQ interaction is also required for engulfment efficiency, functioning like a ratchet to promote membrane migration around the forespore. Here, we screened for additional factors required for engulfment using transposon sequencing in Bacillus subtilis mutants with mild engulfment defects. We discovered that YrvJ, a peptidoglycan hydrolase, and the MurA paralog MurAB, involved in peptidoglycan precursor synthesis, are required for efficient engulfment. Cytological analyses suggest that both factors are important for engulfment when the DMP complex is compromised and that MurAB is additionally required when the SpoIIIAH-SpoIIQ ratchet is abolished. Interestingly, despite the importance of MurAB for sporulation in B. subtilis, phylogenetic analyses of MurA paralogs indicate that there is no correlation between sporulation and the number of MurA paralogs and further reveal the existence of a third MurA paralog, MurAC, within the *Firmicutes*. Collectively, our studies identify two new factors that are required for efficient envelop remodeling during sporulation and highlight the importance of peptidoglycan precursor synthesis for efficient engulfment in B. subtilis and likely other endospore-forming bacteria.

## INTRODUCTION

The cell wall of many bacteria is composed of a mesh-like network of glycan strands cross-linked together by short peptides known as peptidoglycan (PG) (1, 2). PG contributes to bacterial cell shape and rigidity and protects the cell from osmotic lysis and environmental stresses (1, 3). Bacteria constantly undergo PG remodeling in the form of PG synthesis and hydrolysis (3). PG remodeling during vegetative cell growth and division has been extensively studied and led to a clearer understanding of how these two seemingly opposing processes are balanced to allow for controlled growth and cell division (3). Another well-studied example of PG remodeling occurs during differentiation of starving bacteria into dormant spores, during a process called sporulation (4–7). In sporulating bacteria such as Bacillus subtilis, sporulation begins with the formation of a polar septum toward one pole of the cell, dividing the cell into a smaller compartment called the forespore, which eventually becomes the spore, and a larger compartment called the mother cell (4, 6). The mother cell membrane then proceeds to migrate around the forespore in a process called engulfment, until the membranes fuse and the forespore is entirely engulfed by the mother cell (4, 6, 8). In this study, we focus on the characterization of additional genes that function in PG remodeling during engulfment.

The mature spore contains two layers of PG: the germ cell wall and the cortex (9). The germ cell wall is produced during engulfment and is chemically equivalent to vegetative PG (9). The cortex is produced after engulfment and differs from the germ cell wall in that it contains fewer stem peptides, is less cross-linked, and contains muramic δ-lactam (10). Importantly, engulfment cannot initiate and progress without the activity of a 3-protein cell wall degradation complex known as the DMP complex (11). These proteins are produced in the mother cell shortly after polar division and localize to the asymmetric septum. SpoIID is a lytic transglycosylase that cleaves glycan strands lacking stem peptides, SpoIIP is an amidase and endopeptidase that removes the stem peptides from the glycan strands, and SpoIIM is a polytopic membrane protein that is thought to function as a scaffold for the two membrane-anchored enzymes (11, 12). This complex thins the cell wall at the asymmetric septum and cleaves the PG at the leading edge of the engulfing membranes, helping to guide the membrane around the forespore (12, 13). In sporulating cells lacking both SpoIID and SpoIIP, engulfment fails to initiate (11, 12, 14). In hypomorphic mutants, membrane migration around the forespore is impaired and asymmetric (12, 15). A fourth protein, SpoIIB, functions to recruit the DMP complex to the polar septum. In the absence of SpoIIB, the DMP proteins are partially mislocalized, resulting in inefficient PG hydrolysis and impaired engulfment (16).

In addition to PG hydrolysis, the synthesis of new PG has been implicated in engulfment (13), although its role remains unclear. Fluorescent tagging of the antibiotic ramoplanin, which binds the peptidoglycan precursor lipid II, suggested that PG synthesis occurs throughout engulfment (17). Other experiments showed that engulfment occurred less efficiently and was slower when sporulating cells were treated with either cephalexin or bacitracin, a penicillin-binding protein (PBP) blocker and a C_55_-isoprenyl pyrophosphate (generates precursor lipids) recycling inhibitor, respectively (13). Furthermore, protein localization experiments using fluorescent protein fusions to various enzymes involved in PG synthesis showed that they could localize at the engulfing membrane (13). Finally, cryo-electron microscopy (cryo-EM) images have also argued that PG synthesis occurs during engulfment (5, 18). While these studies suggest that PG synthesis is required for engulfment, the exact genes that contribute to this process remain unidentified.

In addition to PG remodeling, engulfment also requires membrane synthesis to facilitate migration of the engulfing mother cell membrane, which eventually generates a second membrane around the spore (8, 19). Migration of these membranes is promoted and stabilized by two highly conserved integral membrane proteins, SpoIIQ and SpoIIIAH (20). SpoIIQ is produced in the forespore, SpoIIIAH is made in the mother cell, and these two proteins interact across the septal membranes (21–24). The SpoIIIAH-SpoIIQ interaction is thought to act like a zipper, stabilizing the engulfing membranes and helping to drive engulfment around the spore (24, 25). Importantly, the role of the SpoIIIAH-SpoIIQ zipper appears to be secondary for engulfment progression, only becoming apparent under certain conditions: for example, in sporulating cells where the cell wall has been artificially removed (25).

Here, we report the identification and characterization of additional genes involved in engulfment. We identified these new players by using transposon insertion sequencing (Tn-seq) (26) to screen for mutants that enhanced mild engulfment defects. We report that a putative PG hydrolase, YrvJ, and an enzyme involved in PG precursor synthesis, MurAB, are required for efficient engulfment. Cytological analysis revealed that both factors are important for engulfment when the DMP complex is compromised and that MurAB is additionally required when the SpoIIIAH-SpoIIQ transenvelope ratchet is missing. Collectively, our studies identified two new proteins that promote efficient envelop remodeling during sporulation and highlight the requirement for PG precursor synthesis for efficient engulfment.

## RESULTS

### A synthetic sporulation screen identifies a relationship between *spoIIB* and genes involved in PG synthesis and hydrolysis.

**FIG 1:**
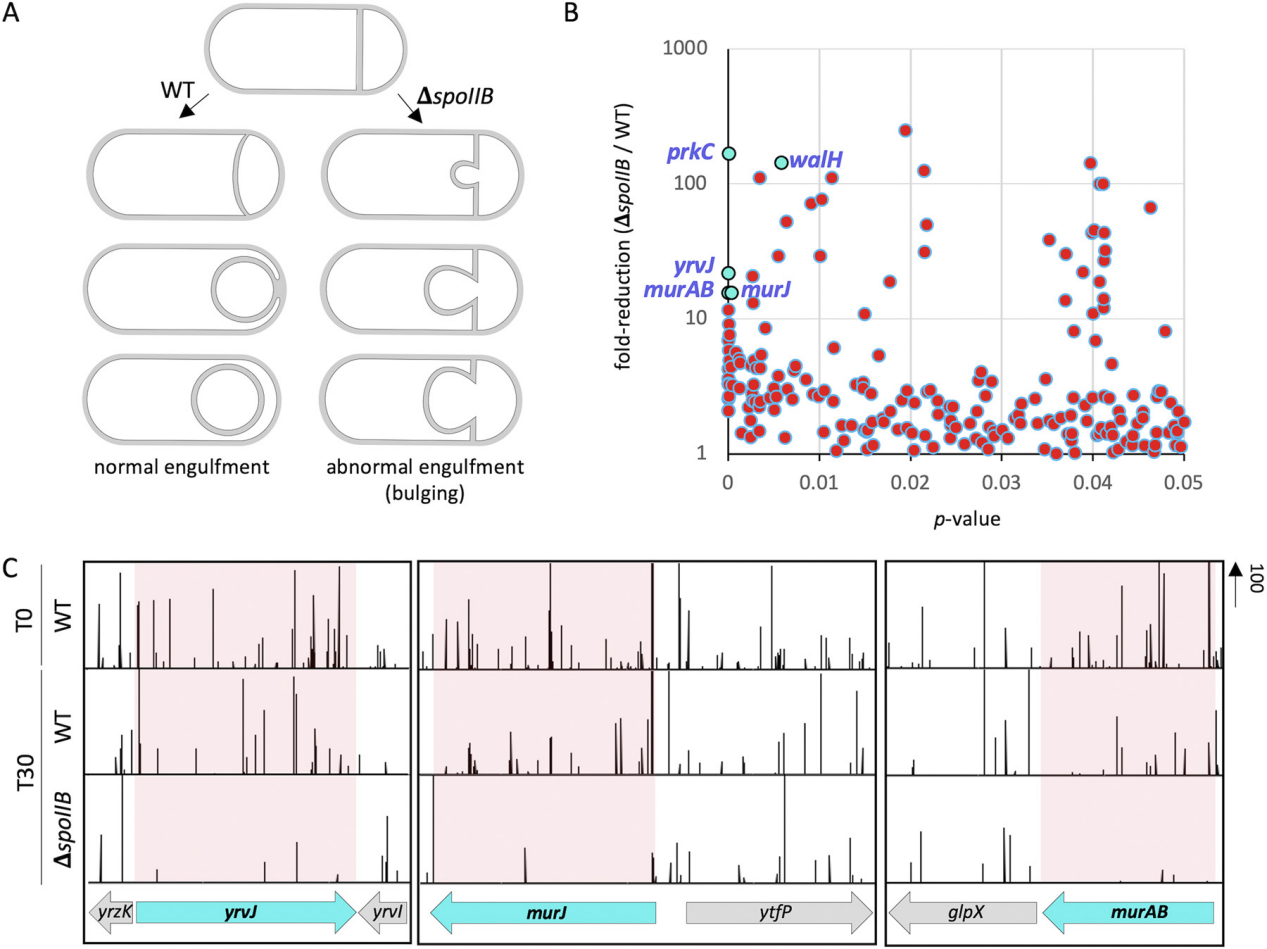
Tn-seq reveals genes involved in PG synthesis and hydrolysis that are important for sporulation in the absence of *spoIIB*. (A) Schematic representation of normal (left) and abnormal (right) engulfment in a wild-type (WT) cell and Δ*spoIIB* mutant cell, respectively. In WT cells, the asymmetric septum curves and engulfment proceeds evenly around the forespore. In Δ*spoIIB* cells, the asymmetric septum bulges and protrudes into the mother cell. PG is shaded in gray. (B) Scatterplot showing fold reduction of transposon insertions in Δ*spoIIB* (bCR1560) relative to WT (bDR2413) cells with corresponding *P* values. Genes involved in PG synthesis (*murAB*, *murJ*) and hydrolysis (*yrvJ*) with high fold reduction in Δ*spoIIB* compared to WT cells and a low *P* value are labeled and colored cyan. (C) Tn-seq profiles at the *yrvJ*, *murJ*, and *murAB* genomic loci of WT (bDR2413) and Δ*spoIIB* (bCR1560) cells following 30 h of growth and sporulation in exhaustion medium. The height of the vertical lines represents the number of Tn-seq reads at each position. Shaded regions highlight the significant reduction in sequencing reads at *yrvJ*, *murJ*, and *murAB* loci.

**FIG 2:**
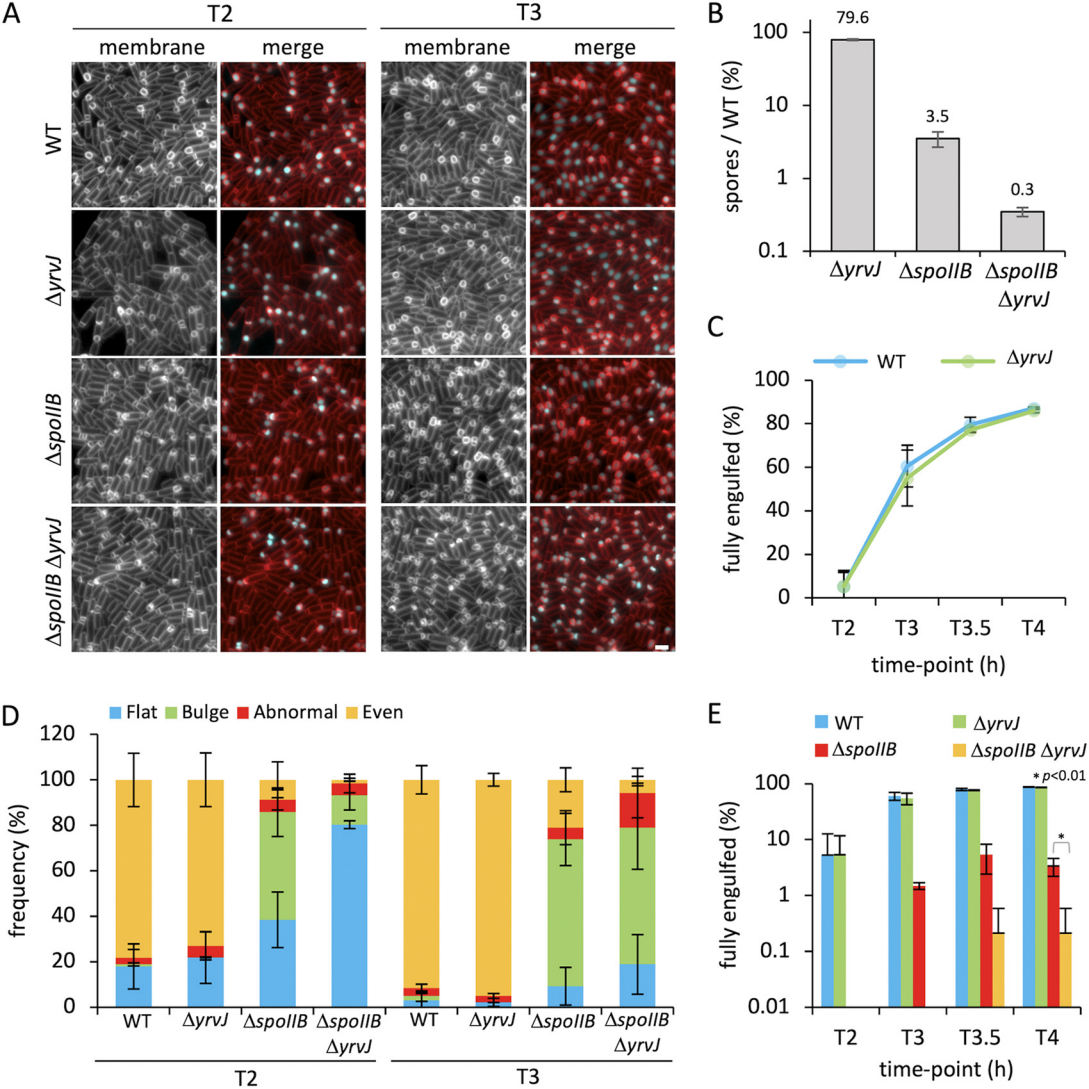
Engulfment initiation and progression in *spoIIB* and *yrvJ* mutants. (A) Engulfment initiation and progression in the wild-type (WT) (bAT68), Δ*yrvJ* (bHC175), Δ*spoIIB* (bHC180), and Δ*spoIIB* Δ*yrvJ* (bHC176) strains at 2 h (T2) and 3 h (T3) after onset of sporulation. Forespore cytoplasm was visualized using a forespore reporter (P*_spoIIQ_*-*cfp* [false-colored cyan in merged images]). Cell membranes were visualized with TMA-DPH fluorescent membrane dye and are false-colored red in merged images. Scale bar = 2 μm. (B) Average sporulation efficiency (mean percentage ± standard deviation [SD]; *n *=* *3) of the Δ*yrvJ* (bAT144), Δ*spoIIB* (bCR1560), and Δ*spoIIB* Δ*yrvJ* (bAT152) mutant strains as a percentage of the WT (bDR2413). Error bars represent SD from three biological replicates. (C) Average frequency (mean percentage ± SD; *n *=* *3) of cells that had completed engulfment during a sporulation time course in WT (bAT68 [blue]) and Δ*yrvJ* (bHC175 [green]) cells, plotted on a nonlogarithmic scale (*n *>* *300 per time point, per strain, per replicate). Error bars represent SD from three biological replicates. (D) Average frequency (mean percentage ± SD; *n *=* *3) of sporulating cells containing flat (blue), bulging (green), abnormal (red), and even (yellow) septa during a sporulation time course in WT (bAT68), Δ*yrvJ* (bHC175), Δ*spoIIB* (bHC180), and Δ*spoIIB* Δ*yrvJ* (bHC176) cells (*n *>* *300 per time point, per strain, per replicate). Error bars represent SD from three biological replicates. Representative images of cells containing each of the septal phenotypes are shown in Fig. S5. (E) Average frequency (mean percentage ± SD; *n *=* *3) of cells that had completed engulfment during a sporulation time course in WT (bAT68 [blue]), Δ*yrvJ* (bHC175 [green]), Δ*spoIIB* (bHC180 [red]), and Δ*spoIIB* Δ*yrvJ* (bHC176 [yellow]) cells (*n *>* *300 per time point, per strain, per replicate). Error bars represent SD from three biological replicates. *, *P* < 0.01 by Student's *t* test performed on the mean of replicates (*n* = 3) at T4 for the Δ*spoIIB* mutant versus Δ*spoIIB* Δ*yrvJ* mutant.

**FIG 3:**
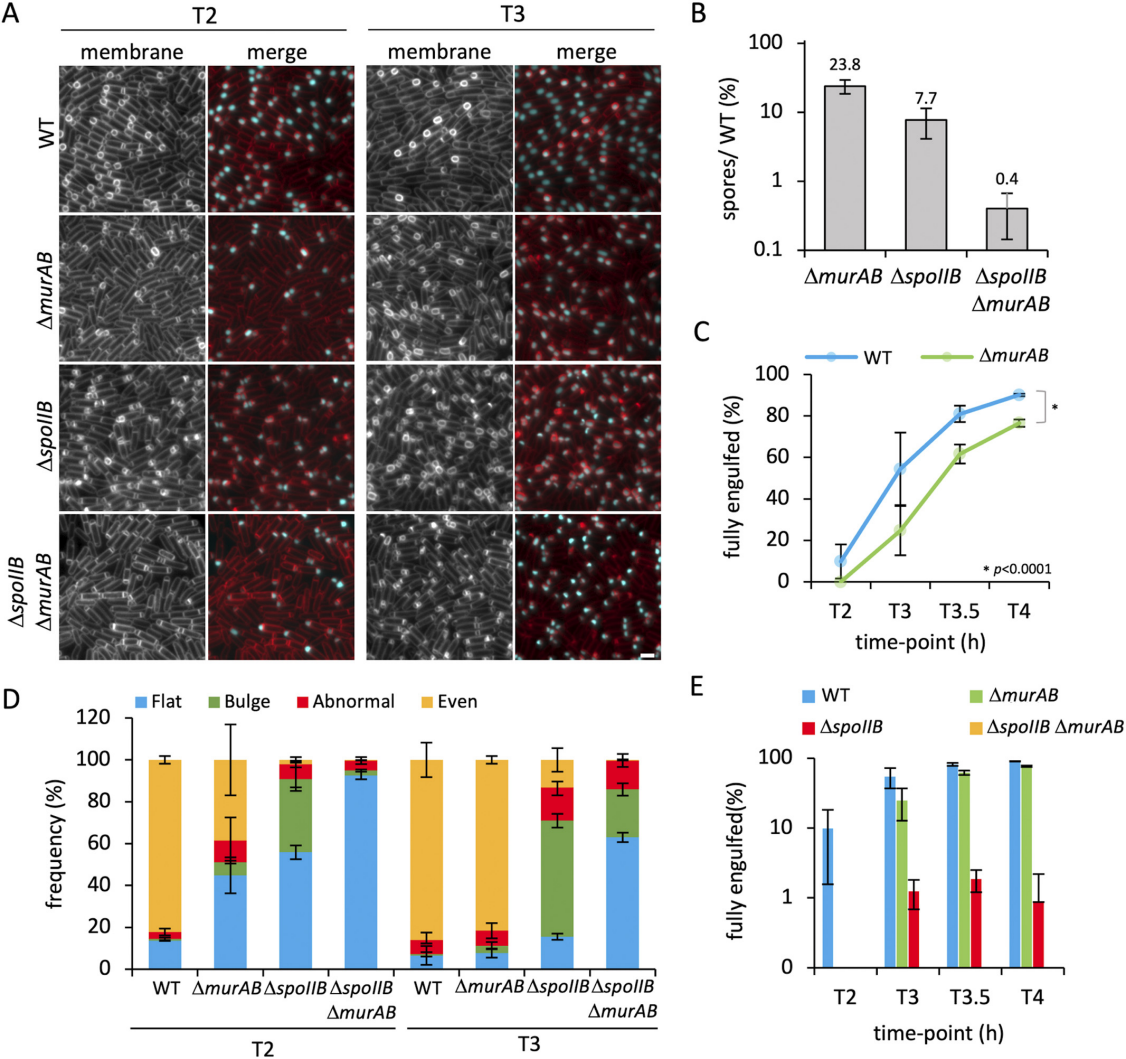
Engulfment initiation and progression in *spoIIB* and *murAB* mutants. (A) Engulfment initiation and progression in the wild-type (WT) (bAT344), Δ*murAB* (bHC217), Δ*spoIIB* (bHC216), and Δ*spoIIB* Δ*murAB* (bHC329) strains at 2 h (T2) and 3 h (T3) after onset of sporulation. Forespore cytoplasm was visualized using a forespore reporter (P*_spoIIQ_*-*gfp* [false-colored cyan in merged images]). Cell membranes were visualized with TMA-DPH fluorescent membrane dye and are false-colored red in merged images. Scale bar = 2 μm. (B) Average sporulation efficiency (mean percentage ± SD; *n *=* *3) of the Δ*murAB* (bAT73), Δ*spoIIB* (bCR1560), and Δ*spoIIB* Δ*murAB* (bHC203) mutant strains as a percentage of the WT (bDR2413). Error bars represent SD from three biological replicates. (C) Average frequency (mean percentage ± SD; *n *=* *3) of cells that had completed engulfment during a sporulation time course in WT (bAT344 [blue]) and Δ*murAB* (bHC217 [green]) cells, plotted on a nonlogarithmic scale (*n *>* *300 per time point, per strain, per replicate). Error bars represent SD from three biological replicates. *, *P* < 0.0001 by Student's *t* test performed on the mean of replicates (*n* = 3) at T4 for the WT versus the Δ*murAB* mutant. (D) Average frequency (mean percentage ± SD; *n *=* *3) of sporulating cells containing flat (blue), bulging (green), abnormal (red), and even (yellow) septa during a sporulation time course in WT (bAT344), Δ*murAB* (bHC217), Δ*spoIIB* (bHC216), and Δ*spoIIB* Δ*murAB* (bHC329) cells (*n *>* *300 per time point, per strain, per replicate). Error bars represent SD from three biological replicates. Representative images of cells containing each of the septal phenotypes are shown in Fig. S3. (E) Average frequency (mean percentage ± SD; *n *=* *3) of cells that had completed engulfment during a sporulation time course in WT (bAT344 [blue]), Δ*murAB* (bHC217 [green]), Δ*spoIIB* (bHC216 [red]), and Δ*spoIIB* Δ*murAB* (bHC329 [yellow]) cells (*n *>* *300 per time point, per strain, per replicate). Error bars represent SD from three biological replicates.

To identify additional factors that contribute to efficient engulfment (Fig. 1A), we used transposon-sequencing (Tn-seq) (26) to screen for genes that become critical for sporulation in cells lacking *spoIIB*. Cells lacking *spoIIB* do not efficiently localize the DMP complex to the septal membrane, resulting in slower and inefficient engulfment and the formation of septal membrane bulges that protrude into the mother cell (27) (Fig. 1A). Furthermore, the Δ*spoIIB* mutant results in 10- to 20-fold fewer heat-resistant spores than the wild type (WT) (27) (Fig. 2B and 3B). We reasoned that cells lacking *spoIIB* are likely sensitized for defects in engulfment and would thus allow the identification of additional genes that function during engulfment.

10.1128/mbio.01732-22.5 FIG S5 Cephalexin reduces SpoIIP levels in sporulating cells. (A) GFP-SpoIIP localization in wild-type (WT) (bHC544) cells treated with and without 50 μg/mL cephalexin for 1 h at 2 h after onset of sporulation (T2). GFP signal is false-colored cyan in merged images. Cell membranes were visualized with TMA-DPH fluorescent membrane dye and are false-colored red in merged images. Scale bar = 2 μm. (B) Immunoblot analysis of SpoIIP in Δ*spoIIP* (bHC550) and WT (bDR2413) cells in the presence (+) and absence (−) of 50 μg/mL cephalexin. Strains were treated for 1 h with cephalexin at T2. SpoIIP was detected using anti-SpoIIP antibodies. Spo0J was used as a loading control and was detected using anti-Spo0J antibodies. Samples from three biological replicates (A, B, and C) are shown. (C) Mean integrated density of SpoIIP bands in cells treated with cephalexin relative to untreated cells as detected by immunoblot analysis in panel B. Error bars represent SD from three biological replicates. *, *P* = 0.0127 by Student’s *t* test. Download FIG S5, TIF file, 2.6 MB. Copyright © 2022 Chan et al. 2022 Chan et al. https://creativecommons.org/licenses/by/4.0/ This content is distributed under the terms of the Creative Commons Attribution 4.0 International license.

10.1128/mbio.01732-22.3 FIG S3 Representative fluorescence microscopy images of engulfment initiation, progression, and completion. (A) Representative membrane fluorescence images of incomplete (left) and complete (right) forespore engulfment in sporulating wild-type (WT) (bAT344) cells. Cell membranes were visualized with TMA-DPH fluorescent membrane dye. Sporulating cells that have not completed engulfment have brighter fluorescent membrane signal intensity around the forespore due to the unfused engulfing membrane, which allows access of the TMA-DPH membrane dye to the entire forespore membrane. Sporulating cells that have completed engulfment have fainter forespore membrane fluorescence due to reduced accessibility of the engulfed forespore to the membrane dye. Scale bar = 2 μm. (B) Representative membrane fluorescence images of flat, bulging, abnormal, and even septa in sporulating WT (bAT344), Δ*spoIIB* (bHC216), Δ*yrvJ* (bHC175), Δ*spoIIB* Δ*yrvJ* (bHC176), Δ*murJ* (bHC218), Δ*spoIIB* Δ*murJ* (bHC230), Δ*murAB* (bHC217), and Δ*spoIIB* Δ*murAB* (bHC329) cells. Cell membranes were visualized with TMA-DPH fluorescent membrane dye. Scale bar = 2 μm. Download FIG S3, TIF file, 2.1 MB. Copyright © 2022 Chan et al. 2022 Chan et al. https://creativecommons.org/licenses/by/4.0/ This content is distributed under the terms of the Creative Commons Attribution 4.0 International license.

Saturated transposon libraries were constructed in the wild type and in the Δ*spoIIB* mutant. At the onset of starvation (T0), a sample was removed from the wild-type culture, and the two cultures were then allowed to exhaust their nutrients and sporulate over the next 30 h (T30). Next, the T30 cultures were incubated at 80°C for 20 min to kill all vegetative and defective sporulating cells and plated on LB agar. Approximately 750,000 colonies originating from spores that had successfully germinated were pooled from each library, and the transposon insertions were mapped by deep sequencing (see Materials and Methods). The insertion profiles in the two libraries after spore formation and outgrowth were compared to each other and the wild-type library at the onset of sporulation.

As expected, transposon insertions in the Δ*spoIIB* mutant were significantly underrepresented in many genes, compared to the wild type (Fig. 1B; see Table S1A in the supplemental material). Some of the genes identified (*murJ* and *murAB*) encode proteins with roles in PG biogenesis (Fig. 1B and C). Furthermore, one of the top hits is a gene that is predicted to encode a PG hydrolase and likely functions as an amidase (YrvJ) (28, 29) (Fig. 1B and C). We took advantage of deletion mutants from the B. subtilis knockout collection (30) to combine mutations in hits from our screen with Δ*spoIIB*. The double mutants were sporulated and analyzed for engulfment defects by fluorescence microscopy. A subset of the hits from the screen enhanced the engulfment defect of the Δ*spoIIB* mutant (Fig. S1A).

10.1128/mbio.01732-22.1 FIG S1 Cytological screen of engulfment phenotype of Δ*spoIIB* double mutant cells and Tn-seq profiles of genes involved in peptidoglycan synthesis and hydrolysis in a SpoIID^T188A^ mutant. (A) Engulfment initiation and progression in Δ*spoIIB* (bHC180) mutant cells and Δ*spoIIB* double mutants with Δ*walH* (bHC459), Δ*xtmA* (bHC460), Δ*tatCY* (bHC461), Δ*fadA* (bHC462), Δ*sigL* (bHC463), Δ*pbpI* (bHC438), Δ*yphA* (bHC464), Δ*yxeR* (bHC465), Δ*ywlB* (bHC469) and Δ*salA* (bHC470) at 3 h after onset of sporulation (T3). Forespore cytoplasm was visualized using a forespore reporter (P*_spoIIQ_*-*cfp* [false-colored cyan in merged images]). Cell membranes were visualized with TMA-DPH fluorescent membrane dye and are false-colored red in merged images. Scale bar = 2 μm. (B) Tn-seq profiles at the *yrvJ*, *murAB*, and *murJ* genomic loci of wild-type (WT) (bDR2413), Δ*spoIIB* (bCR1560), and *spoIID^T188A^* (bCR1574) cells following 30 h of growth and sporulation in exhaustion medium. The height of vertical lines represents the number of Tn-seq reads at each position. Shaded regions highlight the significant reduction in sequencing reads at the *yrvJ*, *murAB*, and *murJ* loci. Download FIG S1, TIF file, 1.0 MB. Copyright © 2022 Chan et al. 2022 Chan et al. https://creativecommons.org/licenses/by/4.0/ This content is distributed under the terms of the Creative Commons Attribution 4.0 International license.

10.1128/mbio.01732-22.10 TABLE S1 List of top 20 Tn-seq hits in the Δ*spoIIB* and Δ*spoIIIAH* mutant. (A) Hits in the Δ*spoIIB* mutant. ^a^Fold difference in the number of transposon insertions: e.g., the Δ*yrvJ* mutant had 21.7-fold fewer transposon insertions in the Δ*spoIIB* than the WT. *, not examined for defects in engulfment by fluorescence microscopy in the Δ*spoIIB* mutant background. (B) Hits in the Δ*spoIIIAH* mutant examined by fluorescence microscopy for defects in engulfment. ^b^Fold difference in the number of transposon insertions: e.g., the Δ*pbpF* mutant had 1,000-fold fewer transposon insertions in the Δ*spoIIIAH* mutant than the WT. Download Table S1, DOCX file, 0.03 MB. Copyright © 2022 Chan et al. 2022 Chan et al. https://creativecommons.org/licenses/by/4.0/ This content is distributed under the terms of the Creative Commons Attribution 4.0 International license.

In a complementary screen, we also conducted a Tn-seq screen in cells harboring a hypomorphic allele of *spoIID* (*spoIID^T188A^*) (12) as the sole source of SpoIID. Similar to the Δ*spoIIB* mutant, the *spoIID^T188A^* allele results in inefficient engulfment and the production of septal membrane bulges, resulting in decreased production of heat-resistant spores relative to WT (~30%) (12). Interestingly, transposon insertions in *murJ*, *murAB*, and *yrvJ* were significantly underrepresented in the *spoIID^T188A^* library compared to the WT (Fig. S1B). Since engulfment involves both synthesis and hydrolysis of PG, we narrowed our focus to these three factors and how they contribute to engulfment in the Δ*spoIIB* mutant.

### YrvJ contributes to efficient engulfment in cells lacking SpoIIB.

Bioinformatic analysis suggests that YrvJ is a secreted amidase with a signal sequence at its N terminus followed by four SH3b domains and a putative amidase domain (28, 29). Interestingly, *yrvJ* is predicted to be expressed under σ^D^ control, and not under the control of a sporulation-specific promoter (31). To investigate this, we fused the *yrvJ* promoter to *gfp* and analyzed fluorescence during sporulation in the wild type and cells lacking σ^D^. As anticipated, the signal was reduced in the absence of σ^D^ (Fig. S2A and B). However, and importantly, *yrvJ* was clearly expressed in wild-type cells during engulfment. To determine the contribution of YrvJ to sporulation, we analyzed sporulation efficiency in the Δ*yrvJ* and Δ*spoIIB* single mutants and the double mutant (see Materials and Methods). In validation of the Tn-seq data, the double mutant produced 0.3% of heat-resistant spores, 10-fold fewer spores than the Δ*spoIIB* mutant (Fig. 2B). Furthermore, consistent with the Tn-seq data in the wild type, the Δ*yrvJ* mutant produced near-wild-type levels of spores (80%) (Fig. 2B). Importantly, the sporulation efficiencies of the Δ*yrvJ* and Δ*spoIIB* Δ*yrvJ* mutants could be complemented to wild-type and Δ*spoIIB* levels, respectively, when *yrvJ* was expressed from the ectopic *ycgO* locus (Fig. S2C).

10.1128/mbio.01732-22.2 FIG S2 Transcriptional regulation of *yrvJ* and complementation of *yrvJ* from an ectopic locus. (A) σ^D^-dependent expression of *gfp* from P*_yrvJ_* in otherwise wild-type (WT) (bHC205) and Δ*sigD* (bHC237) strains. Cell membranes were visualized with TMA-DPH fluorescent membrane dye. Scale bar = 2 μm. (B) Frequency distribution of GFP fluorescence intensity in WT (bHC205) and Δ*sigD* (bHC237) strains shown in panel A. WT and Δ*sigD* cells had average GFP fluorescence intensities (*n* = 3) (yellow dots) of 6 ± 2 and 4 ± 1 arbitrary units (a.u.), respectively (total number of cells, >400 per strain). *, *P* < 0.05 by Student’s *t* test and Kolmogorov Smirnov test. (C) The Δ*yrvJ* phenotype can be complemented by expression of *yrvJ* from an ectopic locus. Shown is the average sporulation efficiency (mean percentage ± SD; *n *=* *3) of the Δ*yrvJ* (bAT144) and Δ*spoIIB* (bCR1560) mutant strains in the presence and absence (bAT152) of *yrvJ* expressed from an ectopic locus and as a percentage of the WT (bDR2413). YrvJ complemented the Δ*yrvJ* and Δ*yrvJ* Δ*spoIIB* phenotypes and restored sporulation efficiency to 102% ± 24% (bHC173) and 3% ± 1% (bHC174) of the WT, respectively. Error bars represent SD from three biological replicates. Download FIG S2, TIF file, 1.9 MB. Copyright © 2022 Chan et al. 2022 Chan et al. https://creativecommons.org/licenses/by/4.0/ This content is distributed under the terms of the Creative Commons Attribution 4.0 International license.

To begin to appreciate the magnitude of the Δ*spoIIB* Δ*yrvJ* double mutant defect, we used fluorescence microscopy to determine if cells lacking both proteins exhibit a more severe morphological defect during development than the Δ*spoIIB* mutant. Sporulating cells were imaged over a time course from 2 h after the onset of asymmetric division (T2) to observe engulfment phenotypes (Fig. S3). No obvious differences in forespore morphology or pattern of mother cell membrane migration around the forespore were observed between the WT and Δ*yrvJ* mutant during the engulfment process, suggesting that YrvJ plays little or no role in engulfment in otherwise wild-type cells (Fig. 2A). In the Δ*spoIIB* Δ*yrvJ* double mutant, however, the importance of YrvJ for efficient engulfment became apparent (Fig. 2A). Broadly speaking, compared to the Δ*spoIIB* mutant, at T2 in the double mutant, we observed a higher proportion of cells with flat septa, whereas at T3, the proportion of cells with flat septa appeared to be similar (Fig. 2A). Quantification of the number of cells with flat septa confirmed these observations (Fig. 2D). At T2, 38% and 80% of cells contained flat septa in the Δ*spoIIB* mutant and Δ*spoIIB* Δ*yrvJ* double mutant, respectively (2-fold more cells with flat septa in the double mutant than in the Δ*spoIIB* mutant) (Fig. 2D). At T3, 9% and 19% contained flat septa in the Δ*spoIIB* mutant and Δ*spoIIB* Δ*yrvJ* double mutant, respectively (2-fold more cells with flat septa in the double mutant compared to the Δ*spoIIB* mutant) (Fig. 2A and D).

In addition to the above phenotypes, at T3 we noticed that the septal bulges formed in the Δ*spoIIB* Δ*yrvJ* double mutant appeared smaller than those in the Δ*spoIIB* mutant (Fig. 2A). Furthermore, the degree of membrane migration around the forespore appeared to be reduced in the Δ*spoIIB* Δ*yrvJ* double mutant relative to the Δ*spoIIB* mutant (Fig. 2A). These observations led us to consider that the double mutant may also be defective in engulfment completion. Indeed, quantification of the number of cells that had completed engulfment over time revealed that by T4, 0.2% of the double mutant cells had completed engulfment, compared to 3% and 87% in the Δ*spoIIB* mutant and wild-type strain, respectively (Fig. 2C and E). Given the predicted function of YrvJ as an amidase, these results suggest that YrvJ contributes to efficient septal PG hydrolysis to promote membrane migration. In the absence of YrvJ, other sporulation-specific hydrolases can perform this function. However, under conditions in which the sporulation hydrolases are crippled, the role of YrvJ in this process is revealed.

### MurAB contributes to efficient engulfment in cells lacking SpoIIB.

MurAB is a paralog of the essential B. subtilis protein MurAA, which functions as a UDP-*N*-acetylglucosamine 1-carboxyvinyltransferase, catalyzing the first committed step of PG precursor synthesis—the conversion of UDP-*N*-acetylglucosamine to UDP-*N*-acetylglucosamine enolpyruvate that precedes formation of UDP-*N*-acetylmuramic acid (Fig. S4C) (32). Unlike MurAA, MurAB is not essential in B. subtilis, and Tn-seq data suggest that MurAB is not critical for sporulation in otherwise wild-type cells (Fig. 1C) (33). However, our data suggest that this enzyme becomes important when engulfment is partially compromised in the Δ*spoIIB* mutant (Fig. 1C). In agreement with the Tn-seq data, the Δ*murAB* single mutant had a modest defect in sporulation, producing 24% heat-resistant spores compared to the wild type (Fig. 3B). However, the Δ*spoIIB* Δ*murAB* double mutant was reduced to 0.4% heat-resistant spores, compared with 8% for the Δ*spoIIB* mutant, representing a 20-fold reduction in sporulation efficiency (Fig. 3B). The reduction in sporulation efficiency could be complemented to wild-type and Δ*spoIIB* levels when *murAB* was reintroduced at its native locus in the Δ*murAB* and Δ*spoIIB* Δ*murAB* mutants, respectively (Fig. S4F).

10.1128/mbio.01732-22.4 FIG S4 Cell width and UDP-MurNAc levels in Δ*murAB* mutants and complementation of *murAB* from an ectopic locus. (A) Fluorescence microscopy images of wild-type (WT) (bHC316) and Δ*murAB* (bHC324) cells expressing GFP in the sporangium (P*_veg_*-*gfp*) at 3 h after the onset of sporulation (T3). Cell membranes were visualized with TMA-DPH fluorescent membrane dye. Scale bar = 2 μm. (B) Frequency distribution of cell width in WT (bHC316) and Δ*murAB* (bHC324) strains calculated using the GFP fluorescence detected in panel A. WT and Δ*murAB* strains had average cell widths (*n* = 3) of 720 ± 130 nm and 681 ± 112 nm, respectively (total numbers of cells, >1,100 per strain). *, *P* < 0.05 by Student’s *t* test and Kolmogorov-Smirnov test. (C) Schematic showing the enzymatic roles of MurAA, MurAB, and MurB in the production of UDP-MurNAc during peptidoglycan precursor synthesis. (D) UDP-MurNAc levels in the WT (bDR2413 [black dots]) and Δ*murAB* (bAT73 [blue dots]) strains relative to the WT as measured by UPLC-MS (see Materials and Methods). Dots represent biological replicates; the horizontal bar represents the mean from three biological replicates (*n *=* *3 per strain). *P* = 0.1821 by Student’s *t* test. (E) Schematic illustration showing the operon in which *murAB* (blue) is located on the B. subtilis genome. Expression of *murAB* is driven by at least three known promoters, including two under σ^A^ control and one under σ^H^ control. (F) Average sporulation efficiency (mean percentage ± SD; *n *=* *3) of the Δ*murAB* (bAT73), Δ*spoIIB* (bCR1560), and Δ*spoIIIAH* (bKH7) mutant strains in the presence and absence (bHC203 and bKH40, respectively) of *murAB* expressed from an ectopic locus and as a percentage of the WT (bDR2413). MurAB partially complemented the Δ*murAB* (bHC272), Δ*spoIIB* Δ*murAB* (bHC299), and Δ*spoIIIAH* Δ*murAB* (bHC300) phenotypes. Error bars represent SD from three biological replicates. Download FIG S4, TIF file, 3.0 MB. Copyright © 2022 Chan et al. 2022 Chan et al. https://creativecommons.org/licenses/by/4.0/ This content is distributed under the terms of the Creative Commons Attribution 4.0 International license.

To determine whether MurAB’s requirement in sporulation was related to a role in engulfment, we examined spore morphogenesis by fluorescence microscopy. Fluorescence microscopy of sporulating cells over time revealed a delay in engulfment initiation in the Δ*murAB* mutant, with 45% of cells having flat asymmetric septa at T2 compared to 14% in wild-type cells (Fig. 3A and D). However, by T3, the Δ*murAB* mutant largely phenocopied the wild type, with the majority of cells exhibiting even migration of the mother cell membrane around the forespore (82% compared to 86% in wild-type cells) (Fig. 3A and D). Consistent with earlier results, at T2, the Δ*spoIIB* mutant had mostly flat septa (56%) or septal membrane bulges (35%); however, in the Δ*spoIIB* Δ*murAB* double mutant, almost all sporulating cells had flat septa (93%) (Fig, A and 3D). At T3, the proportion of Δ*spoIIB* mutants with flat septa had decreased to 16%, with the majority of sporulating cells containing septal membrane bulges (55%) (Fig. 3A and D). In the Δ*spoIIB* Δ*murAB* double mutant, however, the majority of septa remained flat (63%, 4-fold higher than in the Δ*spoIIB* mutant), with a smaller proportion containing septal membrane bulges (23%) (Fig. 3A and D).

The high proportion of Δ*spoIIB* Δ*murAB* double mutant cells with flat septa at T3 indicated that many had not initiated engulfment and raised the possibility that those that had initiated engulfment might be progressing aberrantly. Indeed, when we quantified the proportion of the population that had completed engulfment over time, no Δ*spoIIB* Δ*murAB* mutants had completed engulfment by T4, compared to 0.9% of the Δ*spoIIB* mutant cells and 77% and 90% of the Δ*murAB* mutant and wild-type cells, respectively (Fig. 3C and E). The contribution of Δ*murAB* to the observed engulfment defects in the Δ*spoIIB* Δ*murAB* double mutant is most likely related to a defect in PG synthesis since the Δ*murAB* mutant is significantly thinner than wild-type cells (Fig. S4A and B). Indeed, muropeptide analysis revealed significantly lower levels of UDP-MurNAc (a PG precursor product downstream of MurAB) in Δ*murAB* cells compared to the WT (Fig. S4D). Taken together, these results suggest that MurAB and PG precursor synthesis play a role in efficient engulfment initiation and progression in conditions where PG hydrolysis is compromised during engulfment.

### MurAB contributes to efficient SpoIIP localization.

Our data so far suggest that MurAB contributes to efficient engulfment by playing a role in PG precursor synthesis. Earlier work suggests that PG synthesis is required for efficient localization of the DMP complex to the leading edge of the engulfing membrane during sporulation (13). In this model, newly synthesized PG serves as a substrate for the DMP hydrolases, enabling PG degradation and remodeling of the engulfing membrane to occur. Consistent with this, treatment of sporulating B. subtilis cells with antibiotics (bacitracin and cephalexin) that inhibit PG synthesis led to decreased DMP localization at the engulfing membrane (13).

**FIG 4:**
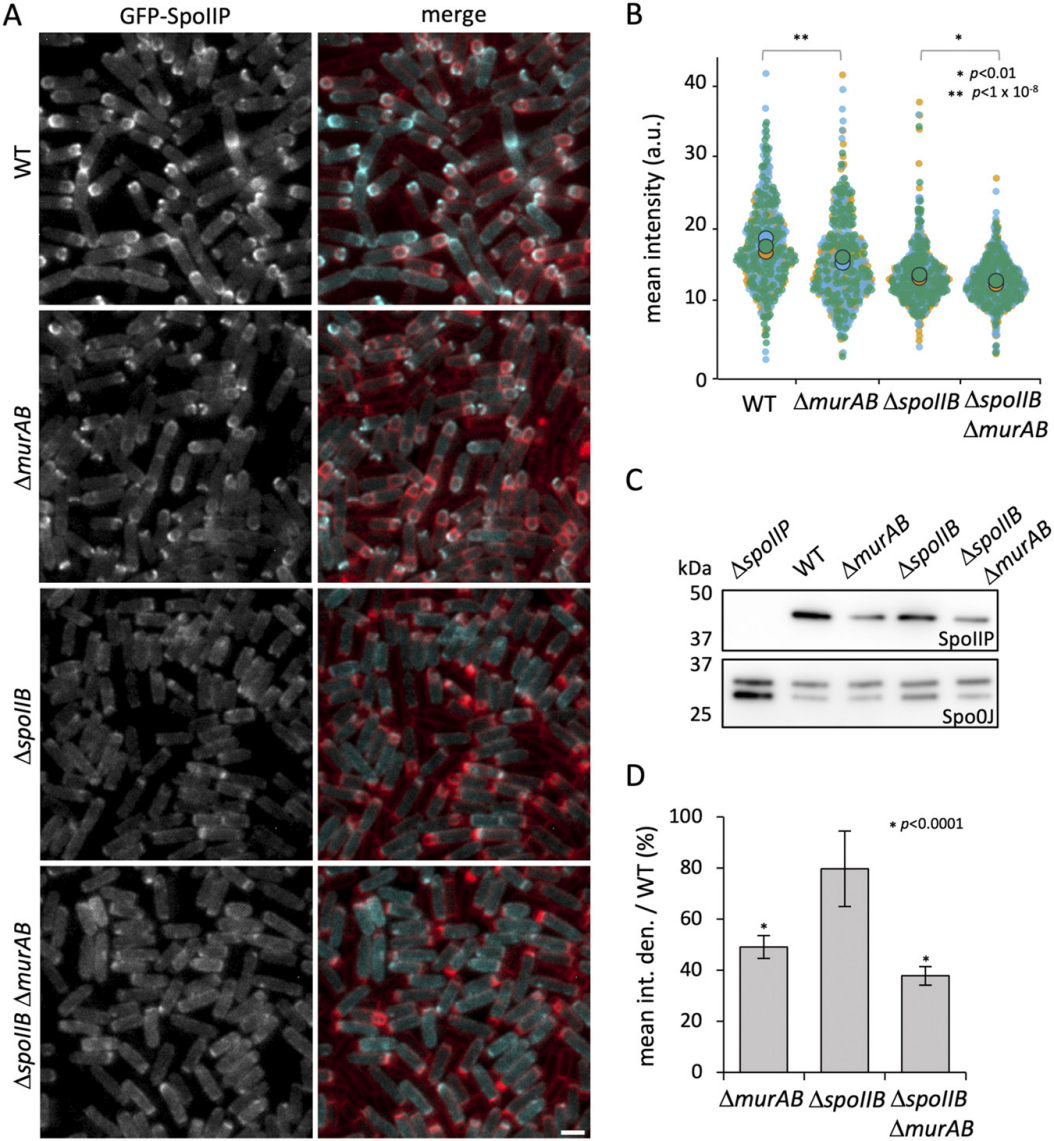
GFP-SpoIIP localization in the absence of *spoIIB* and *murAB*. (A) GFP-SpoIIP localization and (B) distribution of GFP-SpoIIP mean fluorescence intensity (a.u.) in the wild-type (WT) (bHC544), Δ*murAB* (bHC545), Δ*spoIIB* (bHC546), and Δ*spoIIB* Δ*murAB* (bHC547) strains at 2.5 h after onset of sporulation (T2.5). GFP signal is false-colored cyan in merged images. Cell membranes were visualized with TMA-DPH fluorescent membrane dye and are false-colored red in merged images. Scale bar = 2 μm. Panel B is a superplot with totals of 489, 528, 645, and 694 cells for the WT, Δ*murAB*, Δ*spoIIB*, and Δ*spoIIB* Δ*murAB* strains, respectively. *, *P* < 0.01, and **, *P* < 1 × 10^−8^, by Kolmogorov-Smirnov test performed on the combined distribution of replicates (*n* = 3). The Welch’s *t* test was also performed on the means with *P* < 0.05 for the WT versus the Δ*murAB* mutant and the Δ*spoIIB* mutant versus the Δ*spoIIB* Δ*murAB* mutant. (C) Immunoblot analysis of SpoIIP in the Δ*spoIIP* (bHC550), WT (bDR2413), Δ*murAB* (bAT73), Δ*spoIIB* (bCR1560) and Δ*spoIIB* Δ*murAB* (bHC203) strains at T2.5. SpoIIP was detected using anti-SpoIIP antibodies. Spo0J was used as a loading control and was detected using anti-Spo0J antibodies. (D) Mean integrated density of SpoIIP bands relative to the WT as detected by immunoblot analysis in panel C. Error bars represent the SD from three biological replicates. *, *P* < 0.0001 by Student's *t* test.

Because our data are consistent with a role of MurAB in PG precursor synthesis, we wondered whether the engulfment defect in Δ*murAB* and Δ*spoIIB* Δ*murAB* mutants was due to mislocalization of the DMP complex caused by inefficient PG synthesis. To test this, we determined the localization of green fluorescent protein (GFP)-SpoIIP in WT and Δ*spoIIB* mutant backgrounds, in the presence and absence of *murAB*, at 2.5 h after the onset of sporulation (T2.5), after engulfment initiation but before engulfment completion for most cells (see Fig. 3D and E). As previously reported, in wild-type cells, GFP-SpoIIP was present in all mother cell membranes but was enriched at the leading edge of the engulfing membrane (Fig. 4A) (11, 13). The Δ*murAB* mutant had an intermediate phenotype with some enrichment of GFP-SpoIIP at the septal membrane but less than that of the wild type (Fig. 4A). As expected, in the Δ*spoIIB* mutant, GFP-SpoIIP was severely mislocalized from the engulfing membrane (Fig. 4A), as SpoIIB is a localization determinant for SpoIIP and SpoIID (16). The Δ*murAB* Δ*spoIIB* mutant largely phenocopied the Δ*spoIIB* mutant, with virtually no specific enrichment of GFP-SpoIIP at the septum or leading edge of the engulfing membranes (Fig. 4A). Interestingly, the overall GFP-SpoIIP signal appeared reduced in these mutants (Fig. 4A).

To investigate this reduction in GFP-SpoIIP signal, we quantified the average fluorescence intensity in the wild type and the mutants. Our analysis revealed a significant reduction in GFP-SpoIIP fluorescence in Δ*murAB* cells compared to the WT (Fig. 4B) and in Δ*spoIIB* Δ*murAB* cells compared to Δ*spoIIB* cells (Fig. 4B). Furthermore, there was a significant difference in the distribution of average fluorescence intensity of GFP-SpoIIP between WT and Δ*murAB* cells and between Δ*spoIIB* and Δ*spoIIB* Δ*murAB* cells (Kolmogorov-Smirnov test) (Fig. 4B). Immunoblot analysis of SpoIIP levels revealed that the absence of *spoIIB* resulted in a modest but reproducible reduction in SpoIIP levels, with Δ*spoIIB* cells having 80% of wild-type SpoIIP levels (Fig. 4C and D). Interestingly, strains lacking *murAB* had 49% of wild-type SpoIIP levels, and cells lacking both *murAB* and *spoIIB* had 38% of wild-type SpoIIP levels (Fig. 4C and D). Together, our results show that the absence of *murAB* reduces the enrichment of GFP-SpoIIP at the leading edge of the engulfment membrane, as well as the overall fluorescence intensity of GFP-SpoIIP, and the levels of the untagged SpoIIP protein. These data argue that MurAB, and likely PG synthesis, is required for efficient localization and stability of SpoIIP and probably the DMP complex.

To investigate whether MurAB specifically, or PG synthesis more generally, is required for maintaining SpoIIP levels, we treated sporulating cells with the PG synthesis inhibitor cephalexin. Consistent with other published results (13), cells treated with cephalexin had uneven migration of the engulfing membrane around the forespore (Fig. S5A), indicating that PG synthesis had been affected. We then performed immunoblot analysis to compare SpoIIP levels between cephalexin-treated and untreated cells. Our analysis revealed that SpoIIP levels were reduced to 71% ± 11% of WT levels in cells treated with cephalexin (Fig. S5B and S5C). These results support the idea that SpoIIP levels depend on MurAB because of its contribution to PG synthesis.

### MurJ is not required for engulfment in cells lacking SpoIIB.

Our Tn-seq data revealed a third gene involved in PG remodeling, *murJ*, that appears to be more important for sporulation efficiency in the Δ*spoIIB* mutant than in the wild type (Fig. 1C). MurJ transports lipid-linked PG precursors (called lipid II) across the cytoplasmic membrane for their use in cell wall synthesis (34). Sporulation efficiency assays revealed that the Δ*spoIIB* Δ*murJ* double mutant produced 12-fold fewer heat-resistant spores than the Δ*spoIIB* mutant (0.7% and 8%, respectively), whereas the Δ*murJ* mutant was only mildly defective in sporulation, producing 71% heat-resistant spores compared to the wild-type (Fig. S6B). These data validate the Tn-seq and suggest that reduced PG precursors impact sporulation under conditions in which engulfment is impaired.

10.1128/mbio.01732-22.6 FIG S6 Engulfment initiation and progression in *spoIIB* and *murJ* mutants. (A) Engulfment initiation and progression in the wild-type (WT) (bAT344), Δ*murJ* (bHC218), Δ*spoIIB* (bHC216), and Δ*spoIIB* Δ*murJ* (bHC230) strains at 2 h (T2) and 3 h (T3) after onset of sporulation. Forespore cytoplasm was visualized using a forespore reporter (P*_spoIIQ_*-*gfp* [false-colored cyan in merged images]). Cell membranes were visualized with TMA-DPH fluorescent membrane dye and are false-colored red in merged images. Scale bar = 2 μm. (B) Average sporulation efficiency (mean percentage ± SD; *n *=* *3) of the Δ*murJ* (bKH14), Δ*spoIIB* (bCR1560), and Δ*spoIIB* Δ*murJ* (bHC204) mutant strains as a percentage of the WT (bDR2413). Error bars represent SD from three biological replicates. (C) Average frequency (mean percentage ± SD; *n *=* *3) of cells that had completed engulfment during a sporulation time course in WT (bAT344 [blue]), Δ*murJ* (bHC218 [green]), Δ*spoIIB* (bHC216 [red]), and Δ*spoIIB* Δ*murJ* (bHC230 [yellow]) cells (*n *>* *300 per time point, per strain, per replicate). Error bars represent SD of three biological replicates. (D) Average frequency (mean percentage ± SD; *n *=* *3) of sporulating cells containing flat (blue), bulging (green), abnormal (red), and even (yellow) septa during a sporulation time course in WT (bAT344), Δ*murJ* (bHC218), Δ*spoIIB* (bHC216), and Δ*spoIIB* Δ*murJ* (bHC230) cells (*n *>* *300 per time point, per strain, per replicate). Error bars represent SD from three biological replicates. Representative images of cells containing each of the septal phenotypes are shown in Fig. S5. Download FIG S6, TIF file, 3.0 MB. Copyright © 2022 Chan et al. 2022 Chan et al. https://creativecommons.org/licenses/by/4.0/ This content is distributed under the terms of the Creative Commons Attribution 4.0 International license.

Next, we analyzed engulfment in the Δ*spoIIB* Δ*murJ* double mutant using fluorescence microscopy. The mother cell membranes of most Δ*murJ* mutant cells migrated evenly around the forespore during engulfment, at both T2 and T3, similar to wild-type cells (71% and 82%, respectively) (Fig. S6A and D). Surprisingly, engulfment in the Δ*spoIIB* Δ*murJ* double mutant was virtually indistinguishable from that in the Δ*spoIIB* single mutant, with similar proportions of cells containing flat septa at T2 (58% and 52%, respectively) and septal membrane bulges at T3 (53% and 53%, respectively) (Fig. S6A and D). Quantification of the proportion of cells that had completed engulfment over time also did not reveal any detrimental effects of the Δ*murJ* mutation on engulfment completion in the absence of SpoIIB, as both the Δ*spoIIB* and Δ*spoIIB* Δ*murJ* mutants had similar proportions of cells that had completed engulfment by T4 (0.9%) (Fig. S6C). Thus, these data indicate that the contribution of MurJ to efficient sporulation in the Δ*spoIIB* mutant is unrelated to engulfment and therefore likely reflects a distinct role for lipid II flipping during sporulation (see Discussion).

### A synthetic sporulation screen identifies a relationship between *spoIIIAH* and genes involved in PG synthesis.

**FIG 5:**
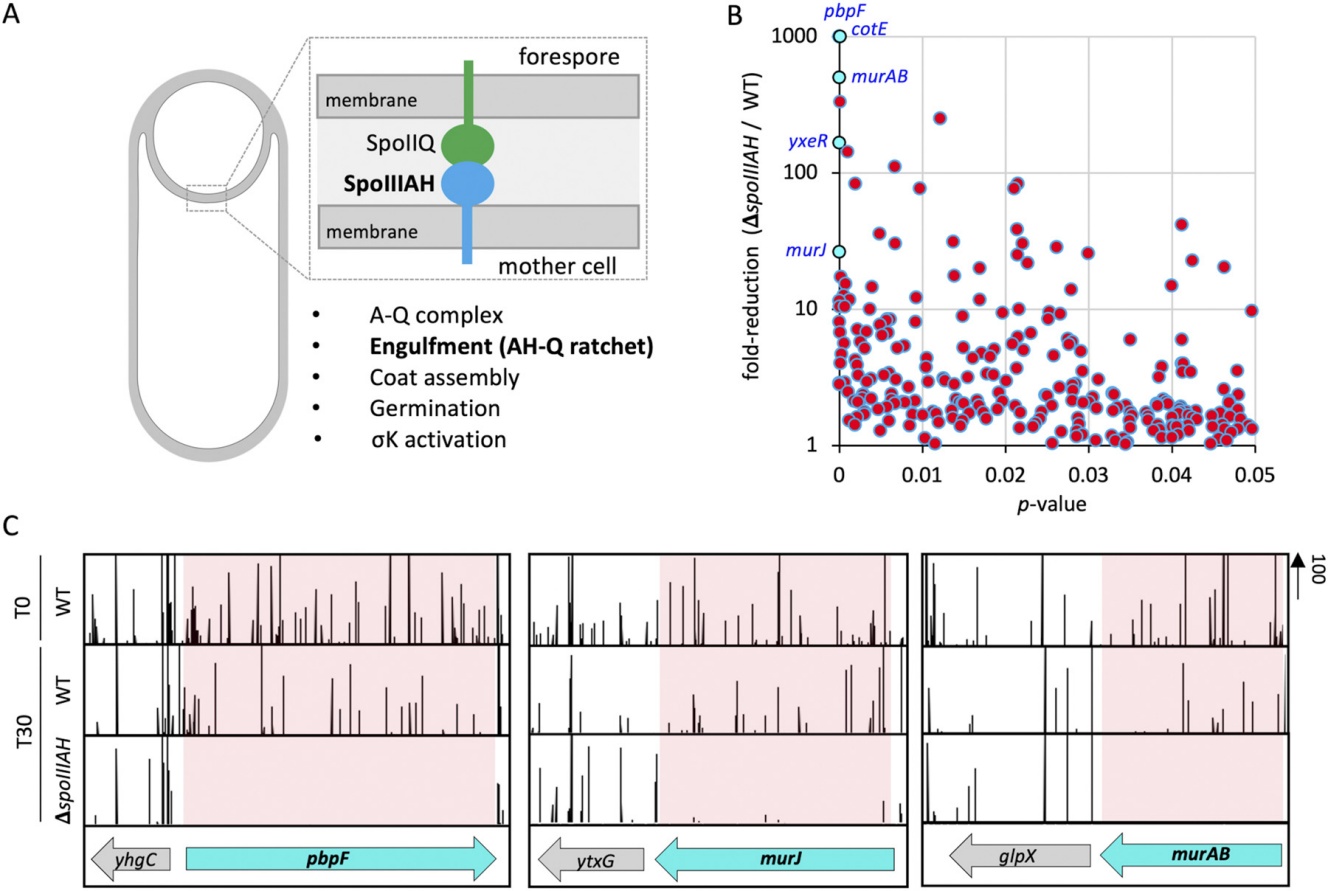
Tn-seq reveals genes involved in PG synthesis and hydrolysis that are important for sporulation in the absence of *spoIIIAH*. (A) Schematic illustration of an engulfing B. subtilis cell (left), highlighting the protein interaction between SpoIIQ (green) in the forespore membrane and SpoIIIAH (blue) in the engulfing mother cell membrane. The SpoIIQ-SpoIIIAH interaction is required for efficient A-Q complex formation, engulfment, coat assembly, germination, and σ^K^ activation. PG is shaded in light gray. (B) Scatterplot showing fold-reduction of transposon insertions in Δ*spoIIIAH* (bCR1117) relative to WT (bDR2413) cells with corresponding *P* values. Genes related to peptidoglycan synthesis (*murAB*, *murJ*, *pbpF*), metabolism (*yxeR*) and coat assembly (*cotE*) with high fold reduction in Δ*spoIIIAH* compared to WT cells and a low *P* value are labeled and colored cyan. (C) Tn-seq profiles at the *pbpF*, *murJ*, and *murAB* genomic loci of WT (bDR2413) and Δ*spoIIIAH* (bCR1117) cells, following 30 h of growth and sporulation in exhaustion medium. Height of vertical lines represents number of Tn-seq reads at each position. Shaded regions highlight the significant reduction in sequencing reads at the *pbpF*, *murJ*, and *murAB* loci.

Our data so far suggest that the Δ*spoIIB* and SpoIID^T188A^ mutants provided sensitized backgrounds to identify additional factors that contribute to engulfment. We wondered whether Tn-seq of a Δ*spoIIIAH* mutant could similarly enable the identification of other factors or pathways that contribute to engulfment. SpoIIIAH is known to function in several morphogenetic pathways during sporulation (e.g., engulfment, assembly of the A-Q complex, localization of the pro-σ^K^ processing complex in the spore membrane, coat assembly) (Fig. 5A) (21, 35–37). Thus, we reasoned that sporulating cells lacking *spoIIIAH* are likely sensitized for defects in all these pathways. To test this, and to identify additional factors that function in engulfment, we performed Tn-seq on a Δ*spoIIIAH* mutant following the same Tn-seq approach described earlier for Δ*spoIIB* and *spoIID^T188A^* (see Materials and Methods).

As expected, transposon insertions in the Δ*spoIIIAH* mutant were significantly underrepresented in many genes compared to the wild type (Fig. 5C and Table S1B). Consistent with SpoIIIAH's role in diverse processes during sporulation, our hits included genes required for the assembly of the coat (e.g., *cotE*, *spoVID*, and *safA*), cortex (*stoA*), and germ cell wall (*pbpF* and *pbpG*) (Fig. 5B and Fig. S7A). Interestingly, *murAB* and *murJ*, identified in the Δ*spoIIB* and SpoIID^T188A^ Tn-seq (Fig. 1B and C), were also identified as top hits in the Δ*spoIIIAH* Tn-seq screen (Fig. 5B and C), raising the possibility that their encoded functions contribute to efficient engulfment in the absence of *spoIIIAH.* Indeed, an initial visual inspection by fluorescence microscopy (Fig. S7) to narrow down genes with synergistic engulfment defects in the Δ*spoIIIAH* mutant, highlighted the need for *murAB* for efficient engulfment (Fig. S7A and B). Specifically, in the Δ*spoIIIAH* Δ*murAB* double mutant, a larger fraction of cells exhibited engulfment defects compared to the Δ*spoIIIAH* single mutant (Fig. S7A and B). We therefore focused on the characterization of this synthetic interaction.

10.1128/mbio.01732-22.7 FIG S7 Cytological screen of engulfment phenotype and σ^G^ activity of Δ*spoIIIAH* cells combined with genetic deletions of top hits identified from Δ*spoIIIAH* Tn-seq screen. Shown are engulfment initiation and progression and σ^G^ activity in (A) Δ*spoIIIAH* (bCR1453) mutant cells and Δ*spoIIIAH* double mutants with Δ*safA* (bCR1496), Δ*ctaG* (bCR1474), Δ*swsB* (bCR1475), Δ*yqgN* (bCR1478), Δ*yumB* (bCR1480), Δ*murJ* (bCR1492), Δ*pbpG* (bCR1493), Δ*pbpF* (bCR1494), Δ*fadN* (bCR1482) and Δ*cotE* (bCR1455) and (B) Δ*spoIIIAH* (bCR1453) mutant cells and Δ*spoIIIAH* double mutants with Δ*ald* (bCR1481), Δ*rnr* (bCR1483), Δ*mscL* (bCR1490), Δ*psd* (bCR1491), Δ*yxeR* (bCR1460), Δ*ytkA* (bCR1457), Δ*murAB* (bCR1458), Δ*rocD* (bCR1485), Δ*ykoY* (bCR1486), and Δ*putR* (bCR1463) at 4 h after onset of sporulation (T4). σ^G^ activity in the forespore was visualized using a σ^G^-dependent forespore reporter (P*_sspB_*-*cfp* [false-colored green in merged images]). Cell membranes were visualized with TMA-DPH fluorescent membrane dye and are false-colored red in merged images. Scale bar = 2 μm. Download FIG S7, JPG file, 1.5 MB. Copyright © 2022 Chan et al. 2022 Chan et al. https://creativecommons.org/licenses/by/4.0/ This content is distributed under the terms of the Creative Commons Attribution 4.0 International license.

### MurAB contributes to efficient engulfment in cells lacking SpoIIIAH.

**FIG 6:**
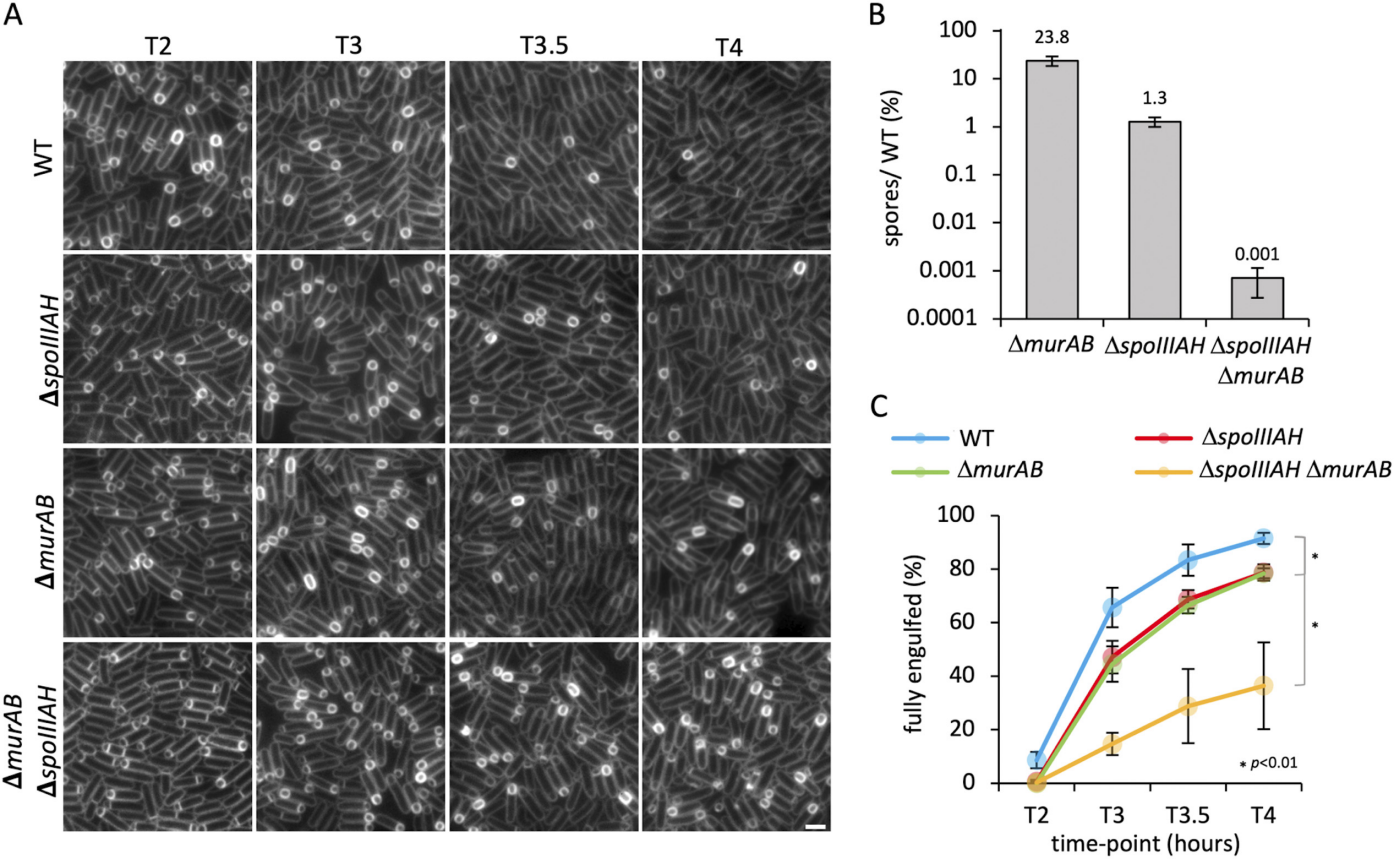
Engulfment initiation and progression in *spoIIIAH* and *murAB* mutants. (A) Engulfment initiation and progression in the wild-type (WT) (bKH3), Δ*spoIIIAH* (bKH4), Δ*murAB* (bKH5), and Δ*spoIIIAH* Δ*murAB* (bKH6) strains at 2 h (T2), T3, T3.5, and T4 after onset of sporulation. Cell membranes were visualized with TMA-DPH fluorescent membrane dye. Scale bar = 2 μm. (B) Average sporulation efficiency (mean percentage ± SD; *n *=* *3) of the Δ*murAB* (bAT73), Δ*spoIIIAH* (bKH7), and Δ*spoIIIAH* Δ*murAB* (bKH40) mutant strains as a percentage of the WT (bDR2413). Error bars represent SD from three biological replicates. (C) Average frequency (mean percentage ± SD; *n *=* *3) of cells that had completed engulfment during a sporulation time course in WT (bKH3 [blue]), Δ*murAB* (bKH5 [green]), Δ*spoIIIAH* (bKH4 [red]), and Δ*spoIIIAH* Δ*murAB* (bKH6 [yellow]) cells (*n *>* *100 per time point, per strain, per replicate). Error bars represent SD from three biological replicates. *, *P* < 0.01 by Student's *t* test performed on the mean of replicates (*n* = 3) at T4 for the WT versus the Δ*murAB* mutant, the WT versus the Δ*spoIIIAH* mutant, and the WT versus the Δ*spoIIIAH* Δ*murAB* mutant.

First, we investigated the sporulation efficiency of the Δ*spoIIIAH* Δ*murAB* double mutant compared to the Δ*spoIIIAH* mutant. Consistent with previous data, the Δ*murAB* mutant produced 24% heat-resistant spores relative to the WT, and the Δ*spoIIIAH* mutant produced 1% (Fig. 6B) (33, 35). However, and in validation of our Tn-seq screen, when the Δ*spoIIIAH* mutant was combined with Δ*murAB*, the double mutant produced only 0.001% heat-resistant spores, corresponding to a 1,000-fold reduction in sporulation efficiency relative to the Δ*spoIIIAH* mutant (Fig. 6B). Reintroduction of *murAB* at its native locus in the Δ*spoIIIIAH* Δ*murAB* double mutant almost fully restored sporulation efficiency to Δ*spoIIIAH* mutant levels (40-fold increase compared to the Δ*spoIIIAH* Δ*murAB*; mutant) (Fig. S4F).

To investigate whether MurAB is also required for efficient engulfment in cells lacking SpoIIIAH, we used fluorescence microscopy to follow engulfment completion over time in the Δ*spoIIIAH* Δ*murAB* and Δ*spoIIIAH* mutants (Fig. 6A). Quantification of the proportion of cells that had completed engulfment revealed that Δ*murAB* and Δ*spoIIIAH* cells completed engulfment at similar rates, albeit with a slight delay compared to the wild type, with 78% and 79% of forespores completely engulfed at T4, compared to 91% of wild-type forespores (Fig. 6C). In the absence of both *spoIIIAH* and *murAB*, however, engulfment completion was severely delayed, and only 36% of cells had completed engulfment at T4 (Fig. 6C). These data provide additional evidence that MurAB contributes to engulfment and is most critical under conditions in which this morphogenetic process is impaired.

### Not all spore-forming bacteria harbor MurAB.

**FIG 7:**
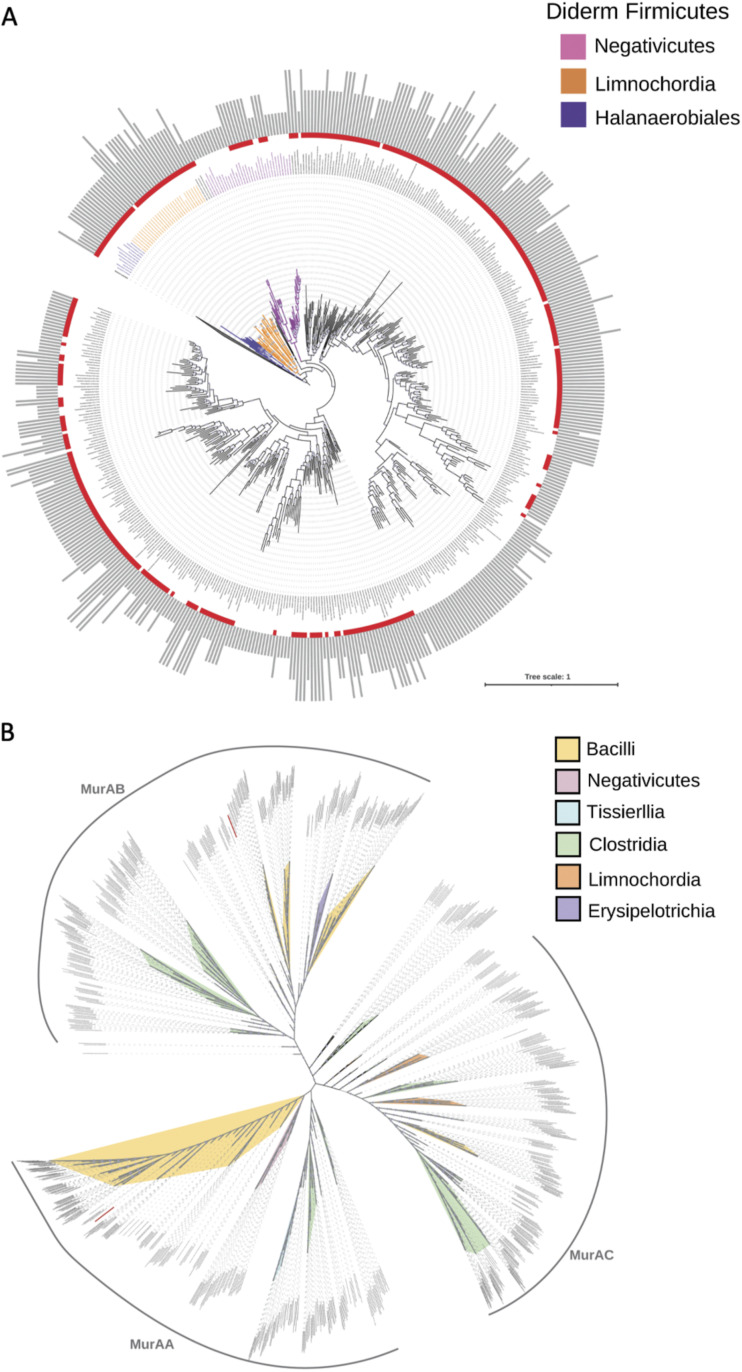
Phylogenetic analysis of MurA homologs. (A) Phylogenetic mapping of MurA (in gray) and Spo0A_C as a marker of sporulation (in red) on a maximum likelihood reference phylogeny of the *Firmicutes* based on a supermatrix containing 497 taxa with 3,776 amino acid positions and inferred with IQ-TREE 1.6.3 using LG+I+G4. Gray dots correspond to supports higher than 80%, and the scale bar corresponds to the average number of substitutions per site. The presence of Spo0A_C homologs is indicated in red in front of each tip. The presence of MurA homologs is indicated with gray bars, and the length of the bar corresponds to the number of paralogs, which varies from 1 to 4. (B) Maximum likelihood phylogenetic tree of MurA homologs from the *Firmicutes* based on an alignment of 938 taxa and 283 amino acid positions and inferred with IQ-TREE using LG+R10. For clarity, only node supports higher than 80% are displayed. The scale bar corresponds to the average number of substitutions per site. A high-resolution version of this figure can be found at https://doi.org/10.6084/m9.figshare.20464098.v1.

The presence of MurAA and MurAB in B. subtilis and our data showing the requirement of MurAB for efficient engulfment (Fig. 3D and E and Fig. 6C) suggest that the presence of MurAB might be a feature of endospore-forming bacteria. However, previous work examining the phylogenetic distribution of MuAA and MurAB based on a reduced taxonomic sample size indicates that this is unlikely to be the case, since Streptococcus pneumoniae, a non-spore-forming bacterium that belongs to the phylum *Firmicutes*, also harbors two MurAs (38). Interestingly, this study also suggested that MurAB is present only in the *Firmicutes* (38). To more comprehensively assess the phylogenetic distribution of MurAA and MurAB in the *Firmicutes* and other bacteria, we conducted two analyses: one on 387 genomes representative of all current bacterial phyla and one on 497 *Firmicutes* genomes. We found that in general, bacterial genomes contain only one MurA paralog. There are very few exceptions with two paralogs, such as one of *Nitrospinae*, one of *Rokubacteria*, and one of *Gemmatimonadetes*. However, these duplications seem specific and do not characterize the whole phyla. In contrast, in the *Firmicutes*, the number of MurA paralogs varies between one and four (Fig. 7A). The phylogeny of all *Firmicutes* MurA paralogs shows that they divide into three clades, MurAA, MurAB, and MurAC (Fig. 7B) as suggested in previous work (39). The number of MurA paralogs is not a specificity of possessing an outer membrane, as the diderm lineages, the *Negativicutes* and the *Halanaerobiales*, have mainly the MurAA paralog, while *Limnochordia*, the third diderm lineage, has mainly the MurAC paralog (Fig. 7B). The majority of *Bacillales* have instead MurAA and MurAB (Fig. 7B). Phylogenetic analysis therefore suggests that MurAA and MurAB might have arisen from an early gene duplication and that MurAB was independently lost afterwards in several lineages. Importantly, we found no correlation between the presence of the sporulation initiation factor Spo0A and the number and type of MurA paralogs (Fig. 7A).

## DISCUSSION

Our study confirms and extends previous work indicating that engulfment requires both new PG synthesis and PG hydrolysis to drive the mother cell membranes around the forespore. Our work uncovered an additional factor YrvJ, a putative amidase, that is not under sporulation control but nonetheless contributes to efficient engulfment. Although cell wall synthesis is also required for engulfment, the specific assembly factors have not been defined, likely due to their essentiality for growth. However, our work indicates that the MurA paralog, MurAB, involved in PG precursor synthesis contributes to efficient engulfment. The roles for both YrvJ and MurAB in engulfment had previously been missed because their mutant phenotypes are relatively modest. However, in combination with mutants in engulfment, they are quite pronounced. Thus, this work also highlights the power of synthetic sporulation screens using Tn-seq.

### YrvJ is only required for sporulation under certain conditions.

Our data suggest that under standard laboratory sporulation conditions, YrvJ is not required for efficient sporulation or engulfment in otherwise wild-type cells (Fig. 2B). However, when engulfment is compromised in the Δ*spoIIB* mutant, YrvJ’s role becomes more important (Fig. 2B and D and Fig. 3E). Given that YrvJ encodes a putative secreted amidase, it is possible that in the absence of proper PG hydrolysis by the DMP complex, YrvJ can compensate for the reduced PG hydrolysis efficiency resulting from a compromised DMP complex. Thus, YrvJ is an additional PG hydrolase that can act during sporulation. Whether YrvJ is specifically recruited to the engulfing membrane and how its expression is regulated under these conditions are not yet known. However, the contribution of YrvJ to engulfment efficiency in the Δ*spoIIB* background raises questions about the role of YrvJ in sporulation under nonstandard conditions. YrvJ appears to be produced in the entire sporangium and is at least partially regulated by the σ^D^ regulon (see Fig. S2A and B in the supplemental material). Other genes regulated by σ^D^ include genes involved in cell wall hydrolysis, cell motility, and biofilm formation (40, 41). This raises the possibility that YrvJ’s role in sporulation is required only under certain conditions, such as promoting sporulation in biofilms. Previous work had identified LytC, a σ^D^-dependent PG hydrolase, as being required for efficient engulfment in engulfment-defective cells (42). Thus, our results with YrvJ support the idea of a connection between expression of σ^D^-dependent genes and the ability of sporulating cells to complete engulfment. The importance of YrvJ for sporulation during biofilm formation remains to be investigated.

### PG precursor synthesis contributes to efficient engulfment.

In this study, we show that Δ*spoIIB* mutants have a more severe sporulation and engulfment defect when combined with a *murAB* deletion (Fig. 3D). MurAB is a paralog of MurAA, an essential enzyme that catalyzes the first committed step in PG precursor synthesis. Consistent with this, we found that Δ*murAB* mutant cells are significantly thinner than wild-type cells (Fig. S4A and B) and result in reproducibly lower levels of UDP-MurNac (Fig. S4D), implicating MurAB in PG precursor synthesis. It had previously been suggested that nascent PG acts as a substrate for the DMP complex, localizing it to the leading edge of the engulfing membrane (13). Based on this reasoning, the worsened engulfment defect in the Δ*spoIIB* Δ*murAB* double mutant might be due to mislocalization of the DMP complex in the absence of nascent PG synthesis ahead of the leading edge of the engulfing membrane. However, we found that even though GFP-SpoIIP was partially delocalized from the leading edge of the engulfing membrane in the absence of MurAB (Fig. 4A), Δ*murAB* cells also had significantly reduced levels of SpoIIP (Fig. 4B to D). We also observed significantly reduced SpoIIP levels in sporulating cells treated with the PG synthesis inhibitor cephalexin (Fig. S5B and C). Together, our results suggest that efficient PG synthesis is required to maintain sufficient SpoIIP levels, which in turn might be required for efficient SpoIIP localization to the leading edge of the engulfing membrane. It is also formerly possible that the absence of MurAB and the addition of cephalexin, as well as the consequent delay in engulfment, trigger a mild degradation of proteins at the engulfing membranes, thereby affecting the stability of SpoIIP (Fig. 4 and Fig. S5) and likely other DMP components.

MurAB was also identified in the Δ*spoIIIAH* Tn-seq screen (Fig. 5B and C). We found that the Δ*spoIIIAH* Δ*murAB* double mutant was severely defective in the formation of heat-resistant spores (Fig. 6B) and exhibits a dramatic delay in engulfment completion (Fig. 6C). We hypothesize that in the Δ*spoIIIAH* mutant, forward movement of the membrane is compromised, and thus when combined with reduced PG precursor synthesis in the absence of *murAB*, the engulfment defect in the double mutant becomes more severe. The engulfment defects observed in the Δ*spoIIIAH* Δ*murAB* double mutant (Fig. 6A and C) are likely attributed to reduced levels and/or mislocalization of the DMP hydrolases due to reduced PG precursor synthesis in the absence of MurAB. That the Δ*spoIIIAH* Δ*murAB* double mutant still completes engulfment in ~1/3 of the population by T4 but nonetheless exhibits a severe defect in the formation of heat-resistant spores suggests that MurAB may contribute to additional stages of spore development (i.e., cortex PG assembly) or even spore germination and outgrowth.

Although our data provide genetic evidence that PG synthesis contributes to engulfment efficiency by two possible routes—(i) maintaining sufficient levels of PG precursors and (ii) promoting stability of DMP proteins—it remains unclear which PG synthases contribute to engulfment. Interestingly, although the two forespore PG synthases PbpF and PbpG, which contribute to germ cell wall synthesis during engulfment, were identified as being important for sporulation in cells lacking Δ*spoIIIAH* (Table S1B), a triple mutant with mutation of all three genes proceeded almost normally throughout engulfment (Fig. S9). This suggests that germ cell wall synthesis by PbpF and PbpG does not contribute to efficient engulfment and that other PG synthases are required for this process. These are possibly essential PG synthases provided by the mother cell or forespore, which would not have been identified by Tn-seq.

10.1128/mbio.01732-22.9 FIG S9 Engulfment initiation and progression of *spoIIIAH* and *pbpF pbpG* mutants. (A) Engulfment initiation and progression in the wild-type (WT) (bDR2413), Δ*spoIIIAH* (bCR1451), Δ*pbpF* Δ*pbpG* (bAT1), and Δ*spoIIAH* Δ*pbpF* Δ*pbpG* (bHC351) strains at 2 h (T2), 3 h (T3), 3.5 h (T3.5), and 4 h (T4) after onset of sporulation. Cell membranes were visualized with TMA-DPH fluorescent membrane dye. Scale bar = 2 μm. (B) Average frequency (mean percentage ± SD; *n *=* *2) of cells that had completed engulfment during a sporulation time course in WT (bDR2413 [blue]), Δ*spoIIIAH* (bCR1451 [green]), Δ*pbpF* Δ*pbpG* (bAT1 [red]), and Δ*spoIIAH* Δ*pbpF* Δ*pbpG* (bHC351 [yellow]) cells (*n *>* *300 per time point, per strain, per replicate). Error bars represent SD from three biological replicates. Download FIG S9, TIF file, 1.6 MB. Copyright © 2022 Chan et al. 2022 Chan et al. https://creativecommons.org/licenses/by/4.0/ This content is distributed under the terms of the Creative Commons Attribution 4.0 International license.

Interestingly, we identified other gene deletions that negatively affected engulfment in the absence of SpoIIB (Fig. S1A). While we chose to focus on genes that have a more direct role in PG synthesis and hydrolysis for this study, other genes may be important for engulfment in the Δ*spoIIB* background, such as *walH* and *prkC* (Fig. S1A). WalH is one of two negative regulators of the WalK sensor kinase, which phosphorylates the DNA-binding WalR and leads to transcription of the WalR regulon, that regulates expression of several genes involved in PG remodeling (43–45). In the absence of WalH, it is tempting to speculate that constitutive expression of these enzymes exacerbates the Δ*spoIIB* mutant defect. PrkC is a serine/threonine kinase which phosphorylates a variety of targets, including RodZ, a putative modulator of MreB filaments (46). Thus, it is possible that in the absence of PrkC, MreB filament assembly is altered, thereby affecting efficient PG synthesis during engulfment. Alternatively, since PrkC is also required for germination (47) and our Tn-seq screens do not distinguish between germination or sporulation mutants, the reduction of transposon insertions in *prkC* in the Δ*spoIIB* mutant background may simply reflect a nonsynergistic genetic interaction that does not worsen the Δ*spoIIB* mutant engulfment defect *per se*, but instead compromises the germination capacity of the few Δ*spoIIB* mutant spores that complete spore maturation. Distinguishing between these possibilities may reveal an additional role of PrkC in the sporulation process.

Finally, although we found that MurJ, a lipid II flippase involved in PG synthesis, is important for efficient sporulation in the Δ*spoIIB* and Δ*spoIIIAH* backgrounds (Fig. S6B and Fig. S8B), it is not required for efficient engulfment in these backgrounds or the conditions tested (Fig. S6C and Fig. S8C). It is therefore likely that MurJ plays a role in sporulation that is not specific to engulfment, but occurs perhaps at later stages of the spore developmental process (i.e., cortex PG synthesis) or spore germination and outgrowth.

10.1128/mbio.01732-22.8 FIG S8 Engulfment initiation and progression of *spoIIIAH* and *murJ* mutants. (A) Engulfment initiation and progression in the wild-type (WT) (bKH21), Δ*spoIIIAH* (bKH23), Δ*murJ* (bKH25), and Δ*spoIIAH* Δ*murJ* (bKH28) strains at 2 h (T2), 3 h (T3), 3.5 h (T3.5), and 4 h (T4) after onset of sporulation. Cell membranes were visualized with TMA-DPH fluorescent membrane dye. Scale bar = 2 μm. (B) Average sporulation efficiency (mean percentage ± SD; *n *=* *3) of the Δ*murJ* (bKH25), Δ*spoIIIAH* (bKH23), and Δ*spoIIIAH* Δ*murJ* (bKH28) mutant strains as a percentage of the WT (bKH21). Error bars represent SD from three biological replicates. (C) Average frequency (mean percentage ± SD; *n *=* *3) of cells that had completed engulfment during a sporulation time course in WT (bKH21 [blue]), Δ*murJ* (bKH25 [green]), Δ*spoIIIAH* (bKH23 [red]), and Δ*spoIIIAH* Δ*murJ* (bKH28 [yellow]) cells (*n *>* *300 per time point, per strain, per replicate). Error bars represent SD from three biological replicates. Download FIG S8, TIF file, 1.7 MB. Copyright © 2022 Chan et al. 2022 Chan et al. https://creativecommons.org/licenses/by/4.0/ This content is distributed under the terms of the Creative Commons Attribution 4.0 International license.

### Phylogenetic distribution of MurAB.

The importance of MurAB and PG synthesis for efficient engulfment raises questions about why B. subtilis cells, and indeed, other members of the *Firmicutes*, encode more than one MurA paralog. Our phylogenetic analyses revealed that multiple MurA paralogs are not specific to spore-forming bacteria (Fig. 7). Instead, the presence of multiple MurA paralogs in some bacteria may be an adaptation to constraints on PG synthesis resulting from environmental or physiological challenges, such as antibiotic exposure. Previous work has suggested that MurAB cannot compensate for the absence of MurAA in B. subtilis (32) but can do so in Streptococcus pneumoniae (38). Thus, in the case of B. subtilis, MurAB may play a specific role in ensuring that additional supplies of PG precursors are funneled into the PG precursor synthesis pathway during engulfment, to ensure efficient PG synthesis during the short period over which engulfment occurs (~1.5 h at 37°C). Consistent with this idea, *murAB* is transcribed in various transcripts (31), one of which is dependent on the primary sigma factor σ^A^ (Fig. S4E), implicated in replenishing housekeeping functions in the mother cell during sporulation (48). Future work investigating MurAC may reveal if it can compensate for the absence of MurAA or MurAB or both.

## MATERIALS AND METHODS

### General methods.

All B. subtilis strains were derived from the prototrophic strain 168 (49), and the oligonucleotide primers used in this study are listed at https://doi.org/10.6084/m9.figshare.20088677.v1. Sporulation was induced by resuspension at 37°C according to the method of Sterlini-Mandelstam (50) or by exhaustion in supplemented Difco sporulation medium (DSM) (51) [8 g/L Bacto nutrient broth (Difco), 0.1% (wt/vol) KCl, 1 mM MgSO_4_, 0.5 mM NaOH, 1 mM Ca(NO_3_)_2_, 0.01 mM MnCl_2_, 0.001 mM FeSO_4_] (52). Sporulation efficiency was determined in 24- to 30-h cultures as the total number of heat-resistant (80°C for 20 min) CFU compared with wild-type heat-resistant CFU. Mutants for the validation of Tn-seq hits were obtained from the B. subtilis Single Gene Deletion Library (Addgene) (30).

### Plasmid construction.

pHC60 [*ycgO*::*PyrvJ-yrvJ* (*spec*)] was generated in a two-way ligation with an EcoRI-HindIII PCR product containing the *yrvJ* promoter and *yrvJ* gene (oligonucleotide primers oAT87 and oAT100 and strain 168 genomic DNA as the template) and pKM083 (*ycgO*::*spec*) cut with EcoRI and HindIII. pKM083 is an ectopic integration vector for double-crossover integration at the nonessential *ycgO* locus (D. Z. Rudner, unpublished data).

### P*_yrvJ_* transcriptional reporter construction.

The *yrvJ::gfp* (*loxP-spec-loxP*) construct was generated by isothermal assembly of PCR products containing a flanking region upstream of the *yrvJ* gene (oligonucleotide primers oHC99 and oHC115 and strain 168 genomic DNA as the template), *gfp* (oligonucleotide primers oHC116 and oHC117 and pAT057 [53] DNA as the template), *loxP*-*spec*-*loxP* (oligonucleotide primers oCR624 and oCR625 and pWX466 DNA as the template), and a flanking region downstream of *yrvJ* (oligonucleotide primers oHC118 and oHC119 and 168 genomic DNA as the template). pWX466 contains the *loxP*-*spec*-*loxP* cassette (Rudner, unpublished).

### Δ*murAB* complementation construction.

The *murAB*::*murAB* (*loxP-spec-loxP*) construct was generated by isothermal assembly of PCR products containing the *murAB* gene and a flanking region upstream of *murAB* (oligonucleotide primers oHC134 and oHC135 and strain 168 genomic DNA as the template), *loxP*-*spec*-*loxP* (oligonucleotide primers oCR624 and oCR625 and pWX466 DNA as the template) and a flanking region downstream of *murAB* (oligonucleotide primers oHC136 and oHC137 and strain 168 genomic DNA as the template). pWX466 contains the *loxP*-*spec*-*loxP* cassette (Rudner, unpublished).

### Transposon insertion sequencing.

Transposon insertion sequencing (Tn-seq) was performed on wild-type (bDR2413), Δ*spoIIB* (bCR1560), *spoIID^T188A^* (bCR1574), and Δ*spoIIIAH* (bCR1117) libraries as described previously (26, 33). Approximately 750,000 transformants were pooled, aliquoted, and frozen. An aliquot was thawed, washed in DSM, and diluted into 50 mL DSM at an optical density at 600 nm (OD_600_) of 0.05. Samples were harvested 24 h later (T24). The T24 samples were incubated at 80°C for 20 min and then plated on LB agar. Approximately 750,000 colonies from germinated spores from each sample were pooled. Genomic DNA was extracted from both samples and digested with MmeI, followed by adapter ligation. Transposon-chromosome junctions were amplified in 16 PCR cycles. PCR products were gel purified and sequenced on the Illumina HiSeq platform using TruSeq reagents (Tufts University TUCF Genomics Facility). Reads were mapped to the B. subtilis 168 genome (NCBI NC_000964.3) and tallied at each TA site, and genes in which reads were statistically underrepresented were identified using the Mann-Whitney U test. Visual inspection of transposon insertion profiles was performed with the Sanger Artemis Genome Browser and Annotation tool.

### Fluorescence microscopy.

Live-cell fluorescence imaging was performed by placing cells on a 2% (wt/vol) agarose pad prepared in resuspension medium and set using a Gene Frame (Bio-Rad). When sporulating cells reached the desired time point, 200 μL of the culture was pelleted by centrifugation and then resuspended in 10 μL of resuspension medium containing the membrane dye TMA-DPH [1-(4-trimethylammoniumphenyl)-6-phenyl-1,3,5-hexatriene *p*-toluenesulfonate] (0.05 mM). After gentle vortexing, 2 μL of the cell suspension was spread on the agarose pad, and a coverslip was placed on top of the gene frame. Cells were imaged by standard epifluorescence using a Zeiss Axioplan 2 microscope equipped with 100× objective NA 1.4. Membrane fluorescence from the TMA-DPH dye was captured using an exposure time of 400 ms. Cyan fluorescent protein (CFP) and GFP images were each acquired with an acquisition time of 800 ms, except GFP-SpoIIP, which had an acquisition time of 2,000 ms.

When required, sporulating cells were treated with 50 μg/mL of cephalexin at T2 (2 h after the onset of sporulation) and then incubated for a further 1 h before preparation for imaging as described above.

### Image analysis and statistics.

Microscopy images were processed by adjusting the brightness and contrast using the Fiji software (54).

Sporulating cells containing flat septa, septal membrane bulges, abnormal migration, and even migration of mother cell membranes around the forespore were manually counted using the Cell Counter plugin in Fiji. Representative examples of cells classified in each of these categories are shown in Fig. S3.

Engulfment completion was determined by the intensity of membrane staining around the forespore. Sporulating cells that have not completed engulfment have brighter fluorescent membrane signal intensity around the forespore due to the unfused engulfing membrane, which allows access of the TMA-DPH membrane dye to the entire forespore membrane (Fig. S3). Sporulating cells that have completed engulfment have fainter forespore membrane fluorescence due to reduced accessibility of the engulfed forespore to the membrane dye (Fig. S3). The proportion of cells that had completed or not completed engulfment was manually counted using the Cell Counter plugin in Fiji.

Cell width measurements and GFP-SpoIIP fluorescence intensity were analyzed using the MicrobeJ plugin (55) designed for the Fiji software. Image background was first subtracted (Process > Subtract Background) to avoid false-positive detection of the fluorescent signal. Next, the “Bacteria” tab on MicrobeJ was set to “Smoothed” to detect the outline of the sporangia from the GFP signal for cell width measurements or from the phase-contrast images for GFP-SpoIIP fluorescence analysis. Three parameters—“Exclude on Edges,” “Shape descriptors,” and “Segmentation”—were checked. For cell width measurements, the generated GFP outlines were further refined by setting the shape descriptors (area, length, width) to correspond to the outlines of individual sporangia. For GFP-SpoIIP fluorescence analysis, the “Maxima” tab on MicrobeJ was set to “Point” to detect fluorescent GFP-SpoIIP foci for measurements of mean fluorescence intensity.

Superplots were generated by inputting MicrobeJ data into the program available at https://huygens.science.uva.nl/SuperPlotsOfData/ (56). A nonparametric Kolmogorov-Smirnov test was used to compare distributions between populations of wild-type and mutant sporulating cells. Welch’s *t* tests were performed to compare the means between populations of wild-type and mutant sporulating cells.

### Immunoblot analysis.

Whole-cell lysates from sporulating cells were prepared as previously described (21). Samples were heated for 5 min at 90°C prior to loading. Equivalent loading was based on OD_600_ at the time of harvest. Samples were separated on a 12.5% (wt/vol) polyacrylamide gel and transferred to a polyvinylidene difluoride (PVDF) membrane. Membranes were blocked in 5% (wt/vol) nonfat milk with 0.5% (wt/vol) Tween 20 for 1 h. Blocked membranes were probed with anti-SpoIIP (1:10,000) (12) or anti-Spo0J (1:5,000) primary antibodies diluted into phosphate-buffered saline (PBS) with 5% (wt/vol) nonfat milk with 0.05%(wt/vol) Tween 20 at 4°C overnight. Primary antibodies were detected with horseradish peroxidase-conjugated anti-rabbit antibodies (Bio-Rad) and detected with Western Lightning ECL reagent as described by the manufacturer.

Band intensities were calculated by measuring the integrated density of bands using Fiji software (54). Integrated density values of SpoIIP bands were normalized based on the integrated density of Spo0J bands from the same strain from the same experiment.

### Phylogenetic analyses.

A local data bank of *Firmicutes* was assembled from the NCBI. First, all the genomes available by April 2020 in the NCBI and annotated as *Firmicutes* were downloaded and dereplicated. Then, we selected one proteome per genus. Proteome selection was realized considering genome characteristics such as assembly level and category. For the assembled *Firmicutes*, the data bank contains 497 genomes. For *Bacteria*, a data bank containing 387 bacterial genomes was assembled, representing all 102 currently available phyla in the NCBI.

In order to build a reference *Firmicutes* phylogeny, exhaustive hidden Markov model (HMM)-based homology searches (with the option –cut_ga) were carried out by using HMM profiles of 34 bacterial ribosomal proteins from the Pfam 29.0 database (57) as queries on the *Firmicutes* data bank using the HMMER-3.1b2 package (58). The retrieved hits of ribosomal proteins were aligned with MAFFT-v7.407 (59) with the auto option and trimmed using BMGE-1.1 (60). The resulting trimmed alignments were concatenated into a supermatrix (497 taxa and 3,776 amino acid positions). A maximum likelihood tree was generated using IQTREE-1.6.3 (61) under the TEST option with 1,000 ultrafast bootstrap replicates.

Homology searches were performed using HMMSEARCH, from the HMMER-3.1b2 package to screen all the proteomes in the *Firmicutes* and *Bacteria* data banks for the presence of MurA homologs. The MurA Pfam domain PF00275.22 and the –cut_ga option were used in the HMMER package. All MurA hits (listed at https://doi.org/10.6084/m9.figshare.20088566.v1) were kept and manually curated using phylogeny, domains, and synteny in order to discard false positives. For MurA hits in the *Firmicutes* data bank, all curated hits were then aligned using CLUSTAL-OMEGA (62) and trimmed with BMGE using default parameters. A maximum likelihood tree was then generated using IQ-TREE version 1.6.12 under the TESTNEW option with 1,000 ultrafast bootstrap replicates. All trees were annotated using IToL (63).

### PG precursor analysis.

Cells were washed three times in ice-cold 0.9% NaCl. The washed pellets were resuspended in 100 μL 0.9% NaCl and boiled for 5 min to lyse the cells and extract the soluble PG precursors. The lysates were centrifuged at 21,000 × *g* for 5 min to pellet the insoluble material, and the resulting supernatant was filtered through a 0.22-μm-pore-size filter for liquid chromatography-mass spectrometry (LC-MS) analysis. Detection and quantification of soluble precursors were performed using an ultraperformance liquid chromatography (UPLC) system (Waters) equipped with an Acquity UPLC BEH C_18_ column (130-Å pore size, 1.7-μm particle size, 2.1 mm by 150 mm; Waters) coupled to a Xevo G2-XS quadrupole time of flight (QTOF) mass spectrometer (Waters). Chromatographic separation of the soluble fraction was performed using a gradient from 0.1% formic acid in water to 0.1% formic acid in acetonitrile over 18 min at 45°C. The QTOF instrument was operated in positive-ion mode, and detection of soluble precursors was performed in the untargeted MS^e^ mode. The MS parameters were set as follows: capillary voltage, 3 kV; source temperature, 120°C; desolvation temperature, 350°C; sample cone voltage, 40 V; cone gas flow, 100 L h^−1^; desolvation gas flow, 500 L h^−1^. Data acquisition and processing were performed using the UNIFI software (Waters). To quantify the soluble precursors, their calculated *m*/*z* ratios were extracted from the total ion current chromatogram, and the corresponding peak in the resulting extracted ion chromatogram was integrated to give a peak area.

### Data availability.

Tn-seq data sets and all materials generated in this work can be made available upon request from the corresponding author, Christopher Rodrigues (christopher.rodrigues@warwick.ac.uk).
