# Supplementary figures and images for: Genetic Screens Identify Additional Genes Implicated in Envelope Remodeling during the Engulfment Stage of Bacillus subtilis Sporulation
Source: mBio. 2022 Sep 6;13(5):e01732-22. doi: 10.1128/mbio.01732-22 (PMC9600426; doi:10.1128/mbio.01732-22)

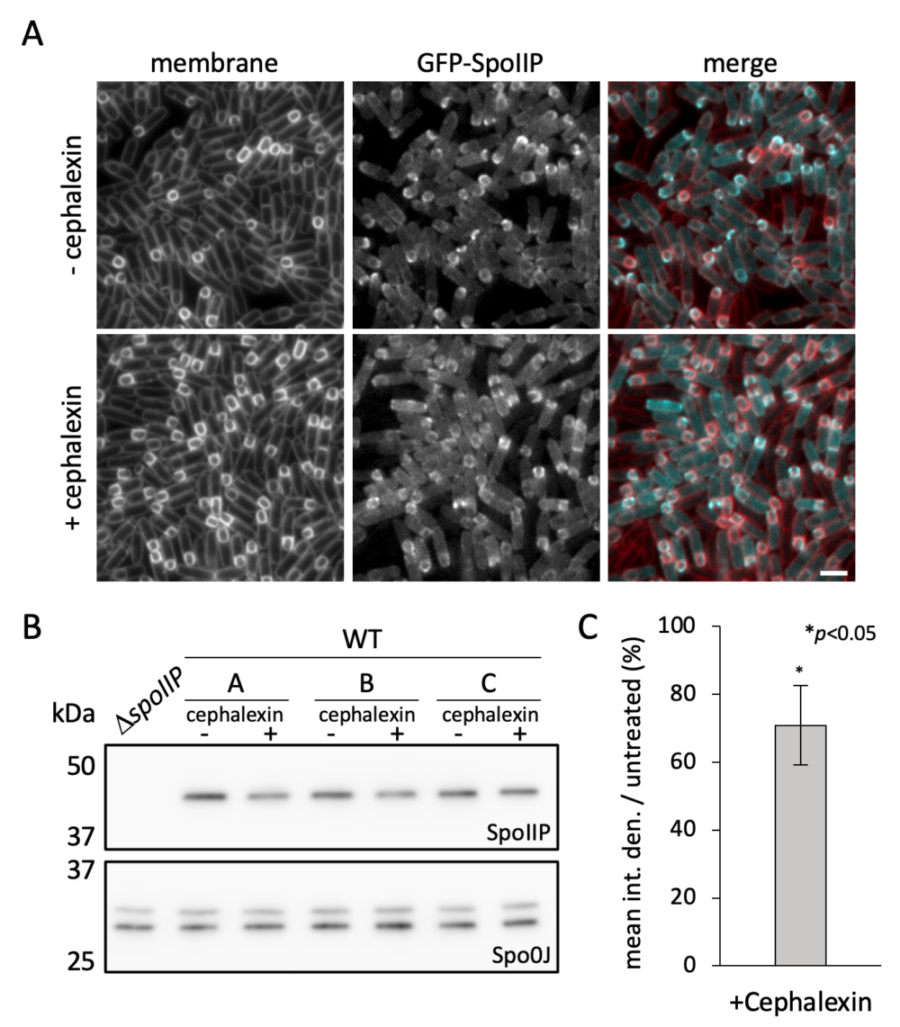

Supplement: FIG S5 [file mbio.01732-22-s0005.tif]

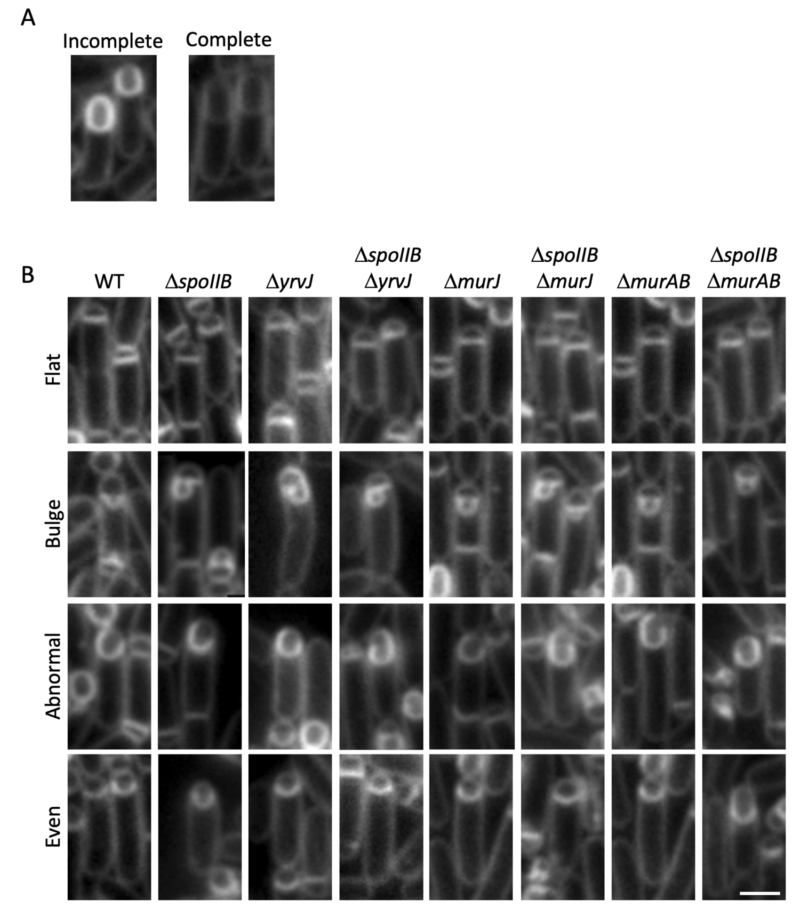

Supplement: FIG S3 [file mbio.01732-22-s0003.tif]

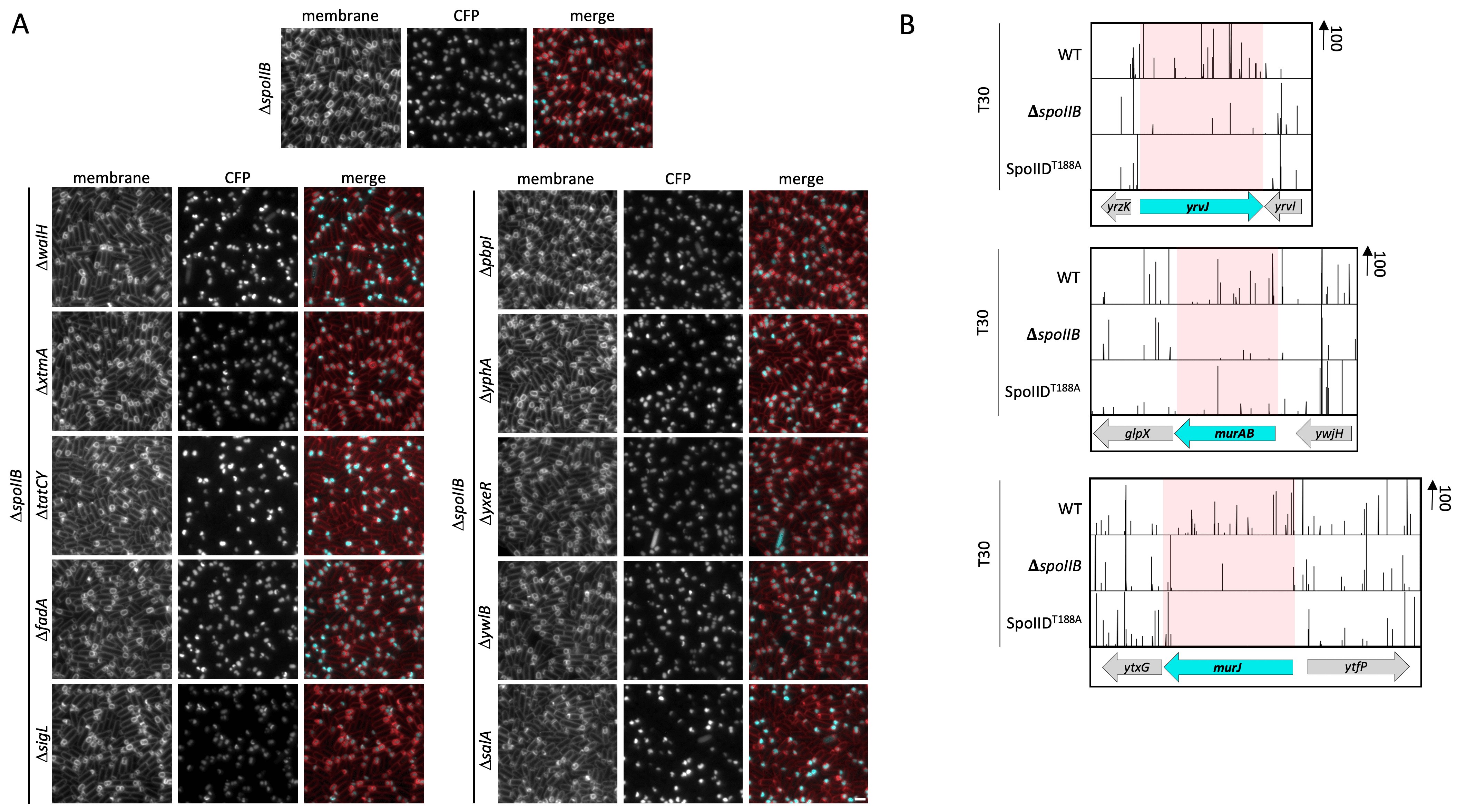

Supplement: FIG S1 [file mbio.01732-22-s0001.tif]

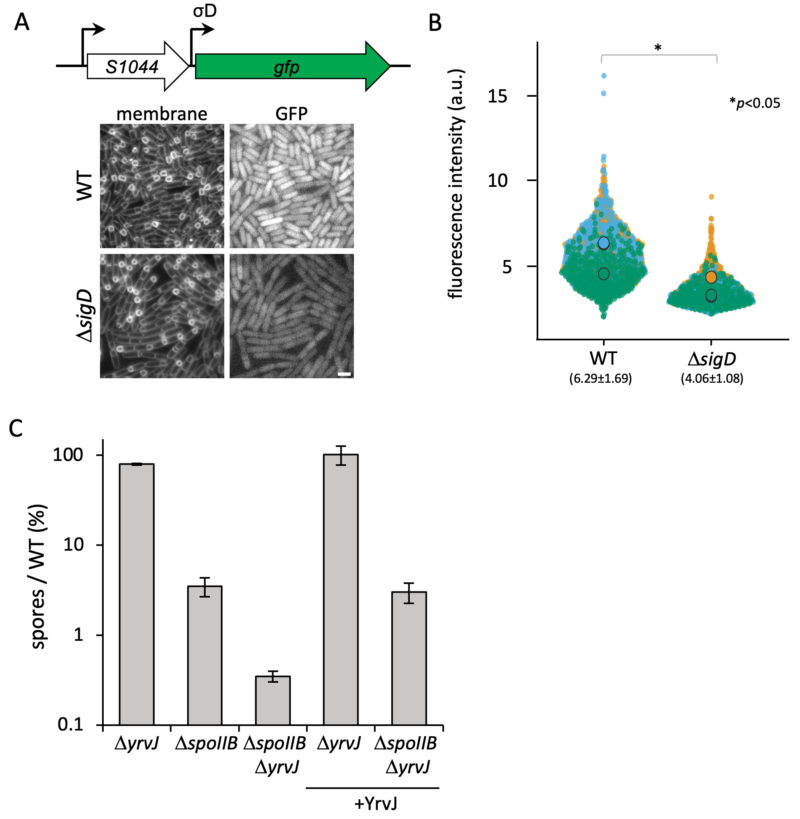

Supplement: FIG S2 [file mbio.01732-22-s0002.tif]

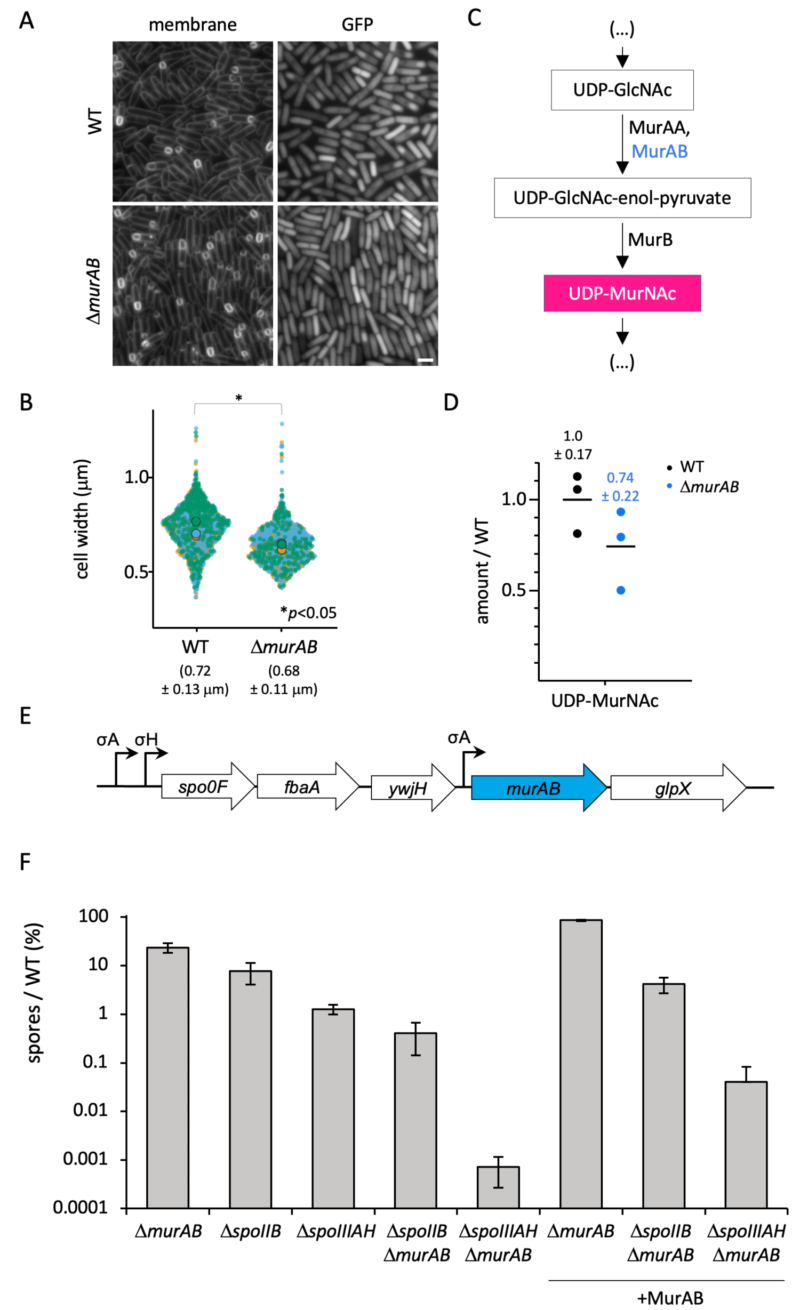

Supplement: FIG S4 [file mbio.01732-22-s0004.tif]

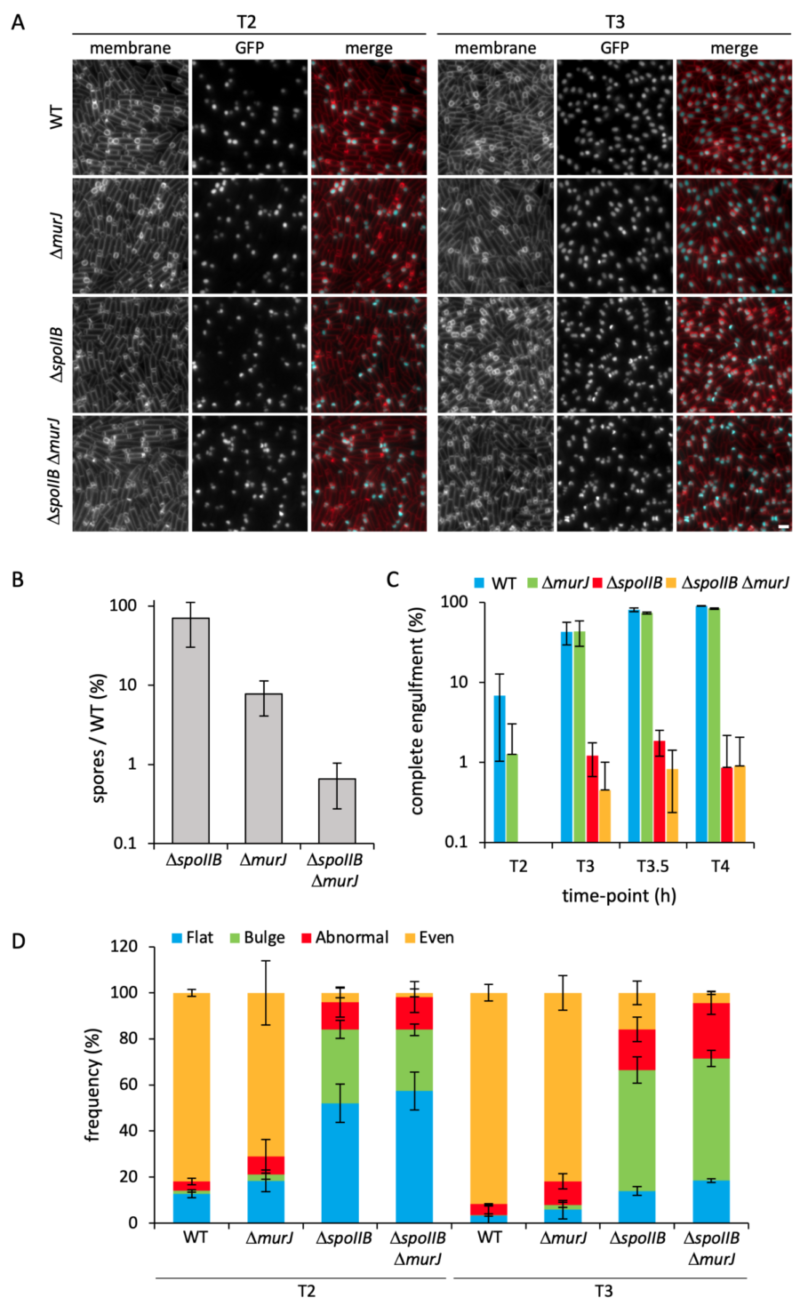

Supplement: FIG S6 [file mbio.01732-22-s0006.tif]

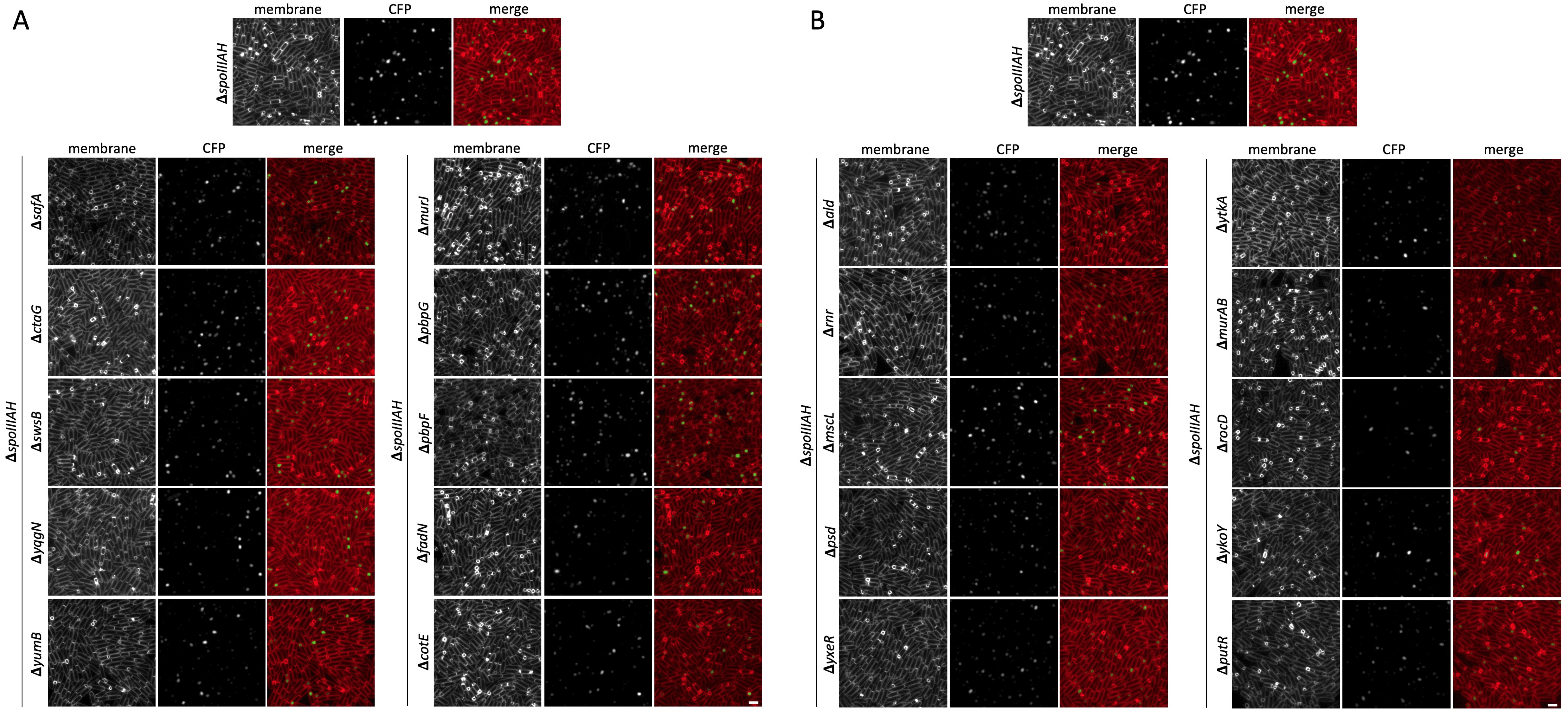

Supplement: FIG S7 [file mbio.01732-22-s0007.jpg]

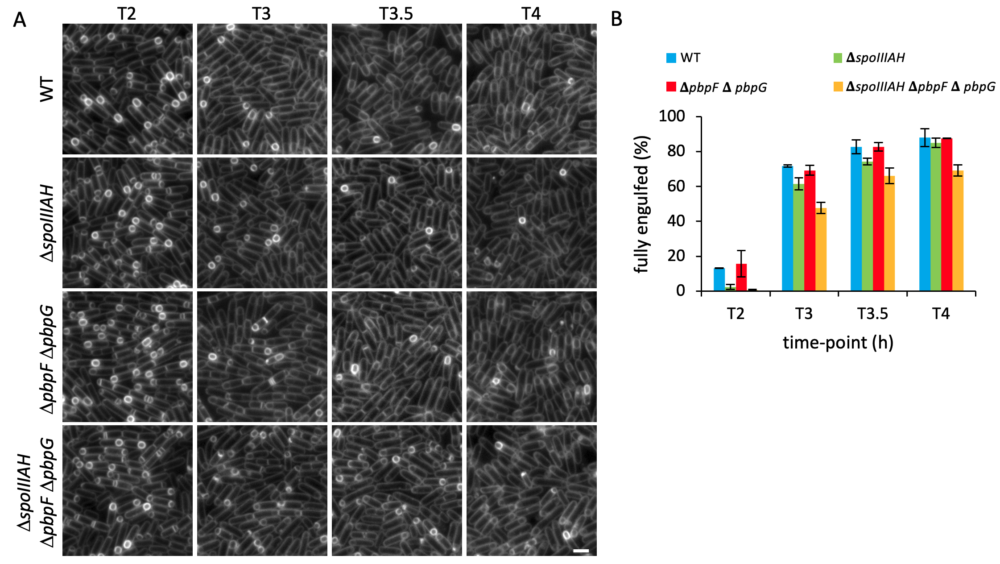

Supplement: FIG S9 [file mbio.01732-22-s0009.tif]

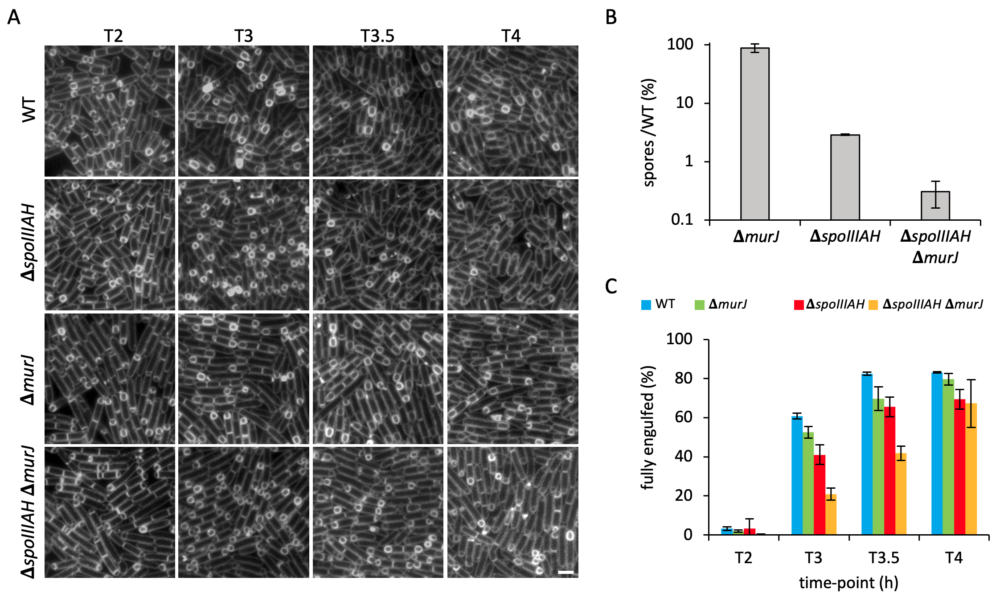

Supplement: FIG S8 [file mbio.01732-22-s0008.tif]
