# Supplementary material for: Genetic Screens Identify Additional Genes Implicated in Envelope Remodeling during the Engulfment Stage of Bacillus subtilis Sporulation
Source: mBio. 2022 Sep 6;13(5):e01732-22. doi: 10.1128/mbio.01732-22 (PMC9600426; doi:10.1128/mbio.01732-22)
Supplement: TABLE S1 [file mbio.01732-22-s0010.docx]

**Table S1. List of top 20 Tn-seq hits in the Δ*spoIIB* and Δ*spoIIIAH* mutant.**

| Table S1A. Hits in the Δ*spoIIB* mutant | | | |
| --- | --- | --- | --- |
| Gene | *p*-value | Tn. fold change ∆*spoIIB* /WT^a^ | Possible function |
| *prkC** | 0.00009 | 166.7 | germination in response to muropeptides |
| *thiO** | 0.00584 | 142.9 | biosynthesis of thiamine |
| *walH* | 0.00343 | 111.1 | control of cell wall metabolism |
| *cwlQ** | 0.00905 | 71.4 | cell wall turnover, lytic transglycosylase |
| *ugtP** | 0.00636 | 52.6 | synthesis of glucolipids and anchoring of lipoteichoic acid, inhibition of [FtsZ](http://www.subtiwiki.uni-goettingen.de/v4/protein?id=41872E2EF00C79918DD077F2EF78F37E24FEB110) assembly |
| *ywlB* | 0.00546 | 29.4 | unknown |
| *yrvJ* | <10^-6^ | 21.7 | cell wall metabolism, peptidoglycan hydrolase |
| *salA* | 0.00264 | 20.8 | control of alkaline protease expression |
| *murJ* | <10^-6^ | 15.6 | export of lipid II, lipid II flippase |
| *murAB* | 0.00033 | 15.6 | peptidoglycan precursor biosynthesis, UDP-N-acetylglucosamine 1-carboxyvinyltransferase |
| *xtmA* | 0.00272 | 13.2 | phage DNA replication |
| *skfC** | <10^-6^ | 11.6 | may be involved in spore killing |
| *pbpI* | 0.00002 | 9.2 | cell wall synthesis, penicillin-binding protein 4b |
| *tatCY* | 0.00403 | 8.5 | TAT [protein secretion](http://subtiwiki.uni-goettingen.de/wiki//index.php/protein%20secretion) |
| *gerAA** | 0.00009 | 7.6 | germination response to L-alanine |
| *fadN* | <10^-6^ | 7.0 | fatty acid degradation |
| *carB** | 0.00005 | 5.8 | biosynthesis of arginine |
| *sigL* | 0.00084 | 5.7 | utilization of arginine, acetoin and fructose, required for cold adaptation |
| *yphA* | 0.00357 | 5.4 | unknown |
| *yxeR* | 0.00117 | 5.1 | ethanolamine uptake/ export |
| \| Table S1B. Hits in the Δ*spoIIIAH* mutant examined by fluorescence microscopy for defects in engulfment \| \| \| \| \| --- \| --- \| --- \| --- \| \| Gene \| *p*-value \| Tn. fold change ∆*spoIIIAH* /WT^b^ \| Possible function \| \| *pbpF* \| 0.000001 \| 1000 \| bifunctional glucosyltransferase/ transpeptidase, synthesis of spore peptidoglycan \| \| *cotE* \| 0.00003 \| 1000 \| assembly of the outer spore coat \| \| *murAB* \| 0.000001 \| 500 \| peptidoglycan precursor biosynthesis \| \| *yxeR* \| 0.000001 \| 166.7 \| ethanolamine uptake/ export \| \| *yqgN* \| 0.00486 \| 35.7 \| unknown \| \| *murJ* \| 0.000001 \| 26.3 \| export of lipid II, peptidoglycan synthesis \| \| *swsB* \| 0.00021 \| 17.2 \| required for spore cortex degradation during [germination](http://subtiwiki.uni-goettingen.de/wiki/index.php/germination) \| \| *rnr* \| 0.00078 \| 15.4 \| nonspecific degradation of rRNA \| \| *mscL* \| 0.00926 \| 12.2 \| resistance to osmotic downshock, glycine betaine export \| \| *putR* \| 0.00004 \| 11.5 \| regulation of proline utilization \| \| *fadN* \| 0.00066 \| 10.4 \| fatty-acid degradation \| \| *ctaG* \| 0.00611 \| 8.4 \| formation of functional cytochrome C-oxidase (caa3) \| \| *rocD* \| 0.00001 \| 8.1 \| arginine, ornithine and citrulline utilization \| \| *ytkA* \| 0.00221 \| 7.1 \| unknown \| \| *yhaX* \| 0.00328 \| 6.8 \| protection of the spore \| \| *yumB* \| 0.00253 \| 5.6 \| unknown \| \| *ald* \| 0.00305 \| 5.2 \| alanine utilization \| \| *ykoY* \| 0.00028 \| 4.7 \| resistance to Mn^2+^ intoxication \| \| *psd* \| 0.00194 \| 4.3 \| biosynthesis of phospholipids \| \| *safA* \| 0.00014 \| 4.0 \| spore coat formation \| \| *pbpG* \| 0.00228 \| 3.3 \| bifunctional glucosyltransferase/ transpeptidase, synthesis of spore peptidoglycan \| | | | |

**^a^** Fold-difference in the number of transposon insertions: e.g. the ∆*yrvJ* mutant had 21.7-fold less transposon insertions in the Δ*spoIIB* than the WT.

**^b^** Fold-difference in the number of transposon insertions: e.g. the ∆*pbpF* mutant had 1000-fold less transposon insertions in the Δ*spoIIIAH* mutant than the WT.

* Not examined for defects in engulfment by fluorescence microscopy in ∆*spoIIB* mutant background.
